# Biomaterial-based local delivery of anti-inflammatory drugs, cytokines, and growth factors for bone regeneration

**DOI:** 10.3389/fbioe.2026.1996624

**Published:** 2026-10-23

**Authors:** Jiaming Shi, Jiahe Liu, Zewen Wang, Haoqiang Wang, Zhaoyang Guo, Baoyi Liu

**Affiliations:** 1 Department of Orthopaedics, Affiliated Zhongshan Hospital of Dalian University, Dalian, China; 2 Dalian Medical University, Dalian, China

**Keywords:** biomaterials, bone defects, bone regeneration, controlled release, growth factors, immunomodulation, local delivery

## Abstract

Large and biologically compromised bone defects remain difficult to repair because structural loss is often accompanied by persistent inflammation, insufficient vascularization, inadequate recruitment of reparative cells, and reduced osteogenic capacity. Biomaterial-based local delivery offers a means of addressing these interacting barriers by retaining therapeutic agents at the defect site, protecting labile bioactive molecules, and controlling the magnitude and duration of local exposure. This review adopts a barrier-oriented and exposure-centered framework that links the dominant biological deficit and stage of healing with therapeutic-agent selection, biomaterial-carrier properties, and local exposure design. We compare major therapeutic categories, scaffold platforms, and strategies for controlling release rate, timing, sequence, spatial distribution, and stimulus responsiveness. Across different bone-defect settings, regenerative performance depends less on maximizing cargo loading, prolonging release, or increasing material complexity than on matching dose, retention, bioactivity, and exposure profile to a defined therapeutic need. Key translational barriers include uncertain dose–exposure relationships, discrepancies between *in vitro* release and *in vivo* exposure, manufacturing and sterilization constraints, reproducibility, and limited clinical validation. Future development should prioritize biologically timed, quantitatively defined, reproducible, and clinically practical delivery systems.

## Graphical Abstract

**Figure:**
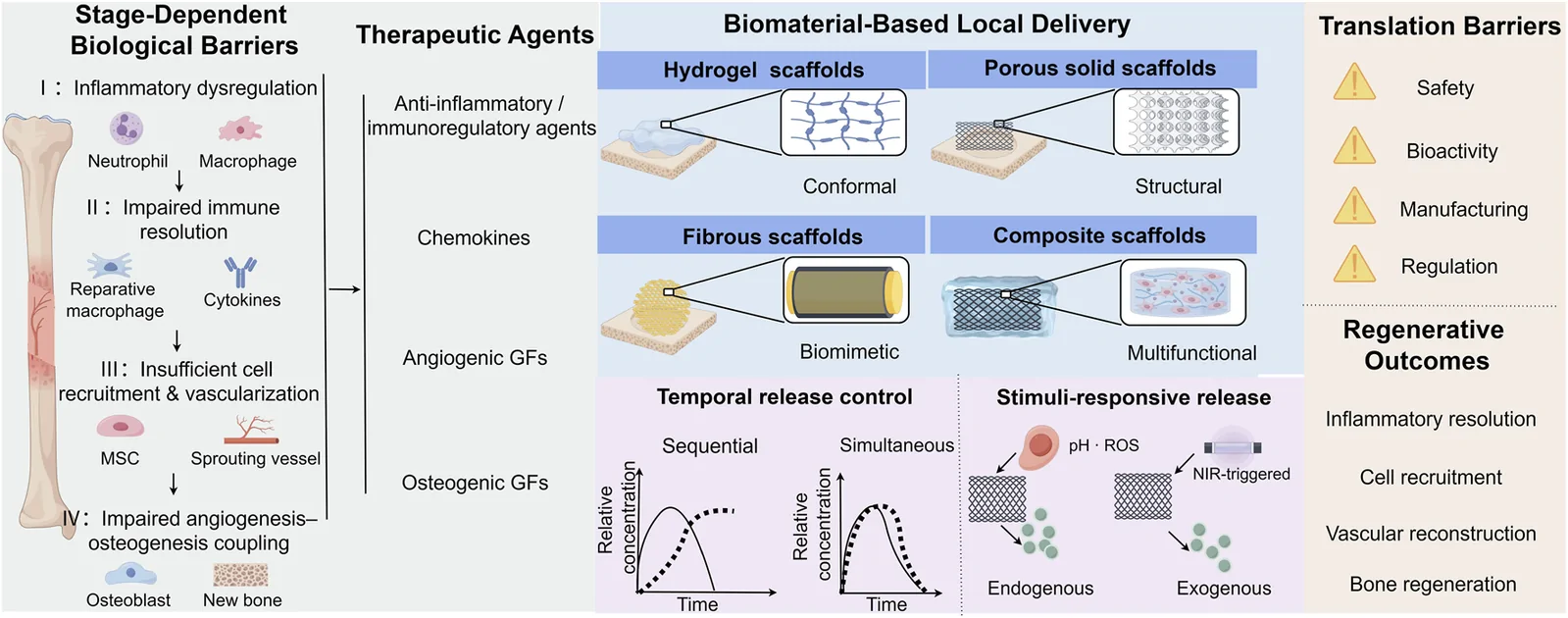
Barrier-oriented framework for biomaterial-based local therapeutic delivery in bone regeneration, linking stage-dependent biological barriers with therapeutic-agent selection, biomaterial delivery platforms, release control, regenerative outcomes, and translational considerations.

## 1 Introduction

Large bone defects resulting from severe trauma, infection-related bone loss, tumor resection, and other pathological conditions remain a major challenge in orthopedic reconstruction (Migliorini et al., 2021). Although bone has substantial intrinsic regenerative capacity, defects that exceed the capacity for spontaneous repair are prone to delayed union or nonunion and often require bone grafting or other reconstructive procedures (Ehlen et al., 2024). Autologous bone grafting remains a mainstay of treatment because it provides osteogenic cells, osteoinductive signals, and an osteoconductive matrix. Its broader use, however, is limited by donor-site morbidity, restricted graft availability, and the need for an additional surgical procedure (Santoro et al., 2025). These limitations have encouraged the development of biomaterial-based strategies for the reconstruction of complex bone defects.

Successful bone repair depends on the coordinated progression of inflammation, angiogenesis, recruitment of reparative cells, osteogenic differentiation, and subsequent remodeling. These processes are closely interconnected and change over the course of healing. An appropriate early inflammatory response contributes to tissue clearance and initiation of repair, whereas persistent or dysregulated inflammation can markedly impair bone regeneration (Molitoris et al., 2024). Angiogenesis is likewise essential because newly forming tissue requires an adequate supply of oxygen and nutrients as well as vascular support for cellular recruitment and function (van Brakel et al., 2024). Large and pathologically compromised bone defects should therefore be viewed not only as structural deficiencies but also as disturbed biological environments in which several reparative processes may be impaired or poorly coordinated.

Anti-inflammatory drugs, cytokines and chemokines, and angiogenic and osteogenic growth factors offer complementary approaches to correcting these local biological deficits, either individually or in combination. Their therapeutic performance after direct local administration, however, may be limited by poor retention, rapid diffusion or degradation, loss of bioactivity, and difficulty in maintaining effective yet safe concentrations. Growth factors illustrate this problem particularly well: their regenerative effects depend not only on the administered dose but also on local retention, preservation of biological activity, and the resulting release profile (Li Y. et al., 2023; Takematsu et al., 2023). Biomaterials can address several of these limitations by retaining and protecting therapeutic agents at the defect site while regulating their local exposure and, in some cases, providing structural support.

Hydrogels, porous solid scaffolds, fibrous scaffolds, and composite systems have all been investigated as local delivery platforms for bone regeneration. Their degradation behavior, diffusional properties, affinity interactions, spatial organization, and responsiveness to biological or external stimuli can be adjusted to control the rate, duration, sequence, and spatial distribution of therapeutic release. No single release profile, however, is optimal for every therapeutic agent or defect. Prolonged release and increased material complexity do not necessarily improve regeneration; the appropriate design instead depends on the properties of the therapeutic cargo, the stage of healing, and the dominant biological barrier within the defect (Szwed-Georgiou et al., 2023; Qi et al., 2024).

Previous reviews have examined biomaterial-based bone regeneration from perspectives including immunomodulatory biomaterials, macrophage-mediated osteoimmunoregulation, growth-factor delivery, and spatiotemporally controlled delivery (Niu et al., 2021; Ball et al., 2023; Li et al., 2025; Liang et al., 2025). Collectively, these reviews provide detailed coverage of individual biological or delivery components, but the relationship among the dominant biological barrier, therapeutic-agent selection, carrier choice, and the required pattern of local therapeutic exposure remains less explicitly integrated. To address this gap, the present review adopts a barrier-oriented and exposure-centered framework that links the biological limitation to be corrected with therapeutic-agent selection, biomaterial-carrier properties, and the required pattern of local therapeutic exposure. We examine anti-inflammatory drugs, cytokines and chemokines, angiogenic and osteogenic growth factors, and functionally complementary therapeutic combinations; compare the major biomaterial delivery platforms and mechanisms used to control local exposure; and consider how these strategies can be tailored to critical-sized, infected, osteoporotic, and diabetes-associated bone defects. We further discuss approaches for evaluating delivery performance and regenerative efficacy, together with the principal barriers to clinical translation. The central premise is that effective local delivery depends on matching the therapeutic agent, carrier, dose, timing, duration of exposure, and spatial localization to the dominant biological deficit and stage of repair, rather than simply maximizing drug loading, extending release, or increasing material complexity. Accordingly, the added value of this review lies not in cataloguing additional agents or biomaterials, but in providing an integrated framework for matching biological barriers with therapeutic agents, biomaterial carriers, and local exposure requirements.

## 2 Biological basis for local therapeutic delivery

The local microenvironment after bone injury evolves continuously during healing, with changes in inflammation, cell recruitment, angiogenesis, and osteogenesis. The dominant biological limitation therefore varies over time: persistent inflammation may predominate at one stage, whereas inadequate vascularization or osteogenesis may become more important at another. These stage-dependent requirements provide the biological basis for selecting therapeutic agents, biomaterial carriers, and release strategies (Jang and Yoon, 2024; Molitoris et al., 2024). The overlapping temporal patterns of pro-inflammatory, anti-inflammatory, pro-angiogenic, and osteogenic signaling during bone healing are summarized schematically in Figure 1A.

**FIGURE 1:**
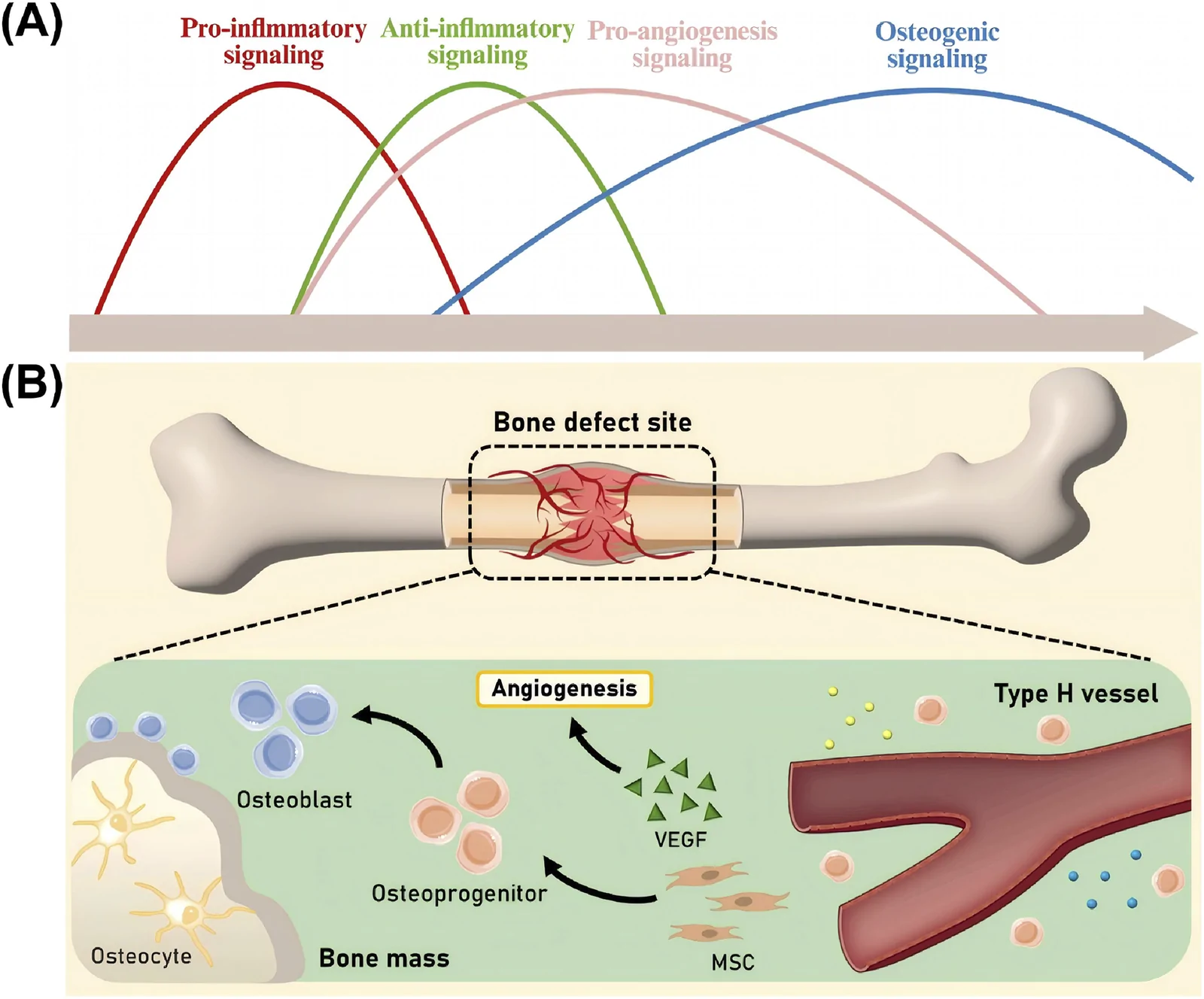
Stage-dependent biological signaling and angiogenesis–osteogenesis coupling during bone repair. **(A)** Schematic temporal overlap of pro-inflammatory, anti-inflammatory, pro-angiogenic, and osteogenic signaling during bone healing. Adapted from Figure 3B of the original study (Yang et al., 2025b). **(B)** Schematic representation of angiogenesis–osteogenesis coupling at a bone-defect site, highlighting interactions among type H vessels, vascular endothelial growth factor (VEGF), mesenchymal stromal cells (MSCs), osteoprogenitors, osteoblasts, and osteocytes. Adapted from Figure 1 of the original study (Jang and Yoon, 2024). The two panels are illustrative schematics adapted from different studies and are not intended for direct quantitative comparison. Source panels were cropped, resized, rearranged, and/or relabeled for the composite layout under the respective Creative Commons Attribution (CC BY) licenses; no underlying experimental data were altered.

Bone injury rapidly produces a hematoma and a hypoxic local environment accompanied by immune-cell infiltration and the release of cytokines and chemokines. Early fracture hematomas contain inflammatory signals together with mediators involved in cell recruitment, angiogenesis, and osteogenesis, indicating that inflammation is an integral component of the early regenerative response rather than a process that should be completely suppressed (Kolar et al., 2011; Hoff et al., 2016; Pountos et al., 2020). Consistent with this view, reducing neutrophil recruitment during early fracture healing suppressed new bone formation and compromised callus mechanical properties (Kovtun et al., 2016). By contrast, persistent inflammation can disrupt the regenerative microenvironment and contribute to nonunion (Hachemi et al., 2024), whereas timely inflammatory resolution improved both bone regeneration and mechanical recovery in aged fracture models (Huang et al., 2020). Effective immunomodulation should therefore preserve the early inflammatory activity required for repair while preventing its persistence or pathological escalation.

As inflammation resolves, vascular reconstruction and bone formation become increasingly important. Angiogenesis supplies oxygen and nutrients to the defect and supports the migration and proliferation of reparative cells. Inhibition of VEGF-mediated angiogenesis markedly impaired callus formation and mineralization, whereas local VEGF delivery promoted vascularization and bone repair (Street et al., 2002). The relationship between angiogenesis and osteogenesis extends beyond nutrient supply: endothelial cells can regulate osteoprogenitor activity, while osteogenic cells in turn influence vascular formation, creating a functional coupling between the two processes during bone healing (Ramasamy et al., 2014; Xu et al., 2018; Jang and Yoon, 2024). This bidirectional angiogenesis–osteogenesis coupling is schematically illustrated in Figure 1B.

Therapeutic priorities therefore shift as healing progresses. Early repair requires sufficient inflammatory activity without uncontrolled or persistent activation, whereas subsequent stages increasingly depend on inflammatory resolution, vascularization, osteogenesis, and remodeling (Yang T. et al., 2025). Large or pathologically compromised bone defects may show persistent disruption of one or more of these processes, providing a rationale for targeted delivery strategies that are matched to the dominant biological barrier and stage of repair. Together, these stage-dependent transitions and angiogenesis–osteogenesis interactions provide the biological rationale for matching therapeutic agents to the dominant repair barrier and temporal requirements of bone healing, as summarized in Figure 1.

## 3 Therapeutic agents for bone regeneration

The choice of therapeutic agent should reflect both the principal biological limitation within the defect and the stage of repair at which that limitation is most relevant. Anti-inflammatory and immunoregulatory agents primarily address persistent or dysregulated inflammation; recruitment-oriented chemokines support reparative-cell recruitment during early-to-intermediate repair; angiogenic factors target insufficient vascular reconstruction as repair progresses; and osteogenic factors address impaired bone formation during later reparative phases. Because these processes overlap temporally, these therapeutic categories should be regarded as stage-informed rather than strictly stage-restricted. When several limitations coexist, agents with complementary functions may be combined, provided that each component addresses a defined regenerative need (Niu et al., 2021; Ball et al., 2023). The major therapeutic categories are compared in Table 1 according to the barriers they are intended to address, their primary delivery goals, and key delivery considerations. To facilitate comparison across therapeutic strategies, the following subsections are organized around the biological problem, therapeutic rationale, delivery requirements, representative evidence, and principal limitations.

**TABLE 1:** Major therapeutic agents and key considerations for local delivery in bone regeneration.

Therapeutic category	Representative agents	Main repair barrier addressed	Primary goal of local delivery	Key delivery considerations	References
Anti-inflammatory and redox-modulating agents	Dexamethasone, aspirin, dimethyl fumarate, quercetin, curcumin	Persistent or excessive inflammation and oxidative stress	Improve the local inflammatory and redox microenvironment without disrupting the inflammatory responses required for normal repair	Control local dose and exposure duration rather than maximizing anti-inflammatory activity	(Su et al., 2023; Hachemi et al., 2024; Huang et al., 2025)
Immunoregulatory cytokines	IL-4, IL-10	Persistent inflammation and immune dysregulation	Maintain local immunoregulatory signaling and promote reparative immune responses	Preserve cytokine bioactivity and maintain an appropriate local concentration and duration of exposure	(Zhang et al., 2020; Li et al., 2022)
Chemokines	SDF-1α (CXCL12), IL-8 (CXCL8)	Insufficient recruitment of reparative cells	Promote migration and recruitment of BMSCs and other reparative cell populations into the defect	Maintain sufficient local chemotactic activity while controlling the magnitude and duration of exposure	(Wang et al., 2024; Zhao et al., 2025)
Angiogenic growth factors	VEGF, FGF-1/2, PDGF-BB, PlGF	Insufficient vascular reconstruction and impaired angiogenesis–osteogenesis coupling	Promote vascularization and establishment of a functional vascular network within the defect	Match local dose and exposure profile to the intended vascular response rather than maximizing vessel formation	(Street et al., 2002; Feng et al., 2026)
Osteogenic growth factors	BMP-2, BMP-7, IGF-1, NELL-1	Insufficient intrinsic osteogenic capacity	Maintain effective local osteogenic stimulation and support new bone formation	Control local dose and exposure, particularly for BMP-2, to limit excessive local stimulation and adverse effects	(Garot et al., 2023; Chernysheva et al., 2025)
Combined therapeutic strategies	VEGF + BMP-2; IL-10 + icariin; SDF-1α + BMP-2; SDF-1α + IGF-1	Coexisting impairments in inflammation, cell recruitment, vascularization, or osteogenesis	Address multiple defined barriers using therapeutically complementary agents	Biological complementarity does not guarantee additional benefit; combined strategies should be compared directly with simpler or single-agent approaches	(Subbiah et al., 2020; Zhou et al., 2021; Li et al., 2024c)

Abbreviations: BMSC, bone marrow mesenchymal stem cell; BMP, bone morphogenetic protein; CXCL, C-X-C motif chemokine ligand; FGF, fibroblast growth factor; IGF-1, insulin-like growth factor-1; IL, interleukin; NELL-1, NEL-like protein-1; PDGF-BB, platelet-derived growth factor-BB; PlGF, placental growth factor; SDF-1α, stromal cell-derived factor-1α; VEGF, vascular endothelial growth factor.

### 3.1 Anti-inflammatory drugs

Persistent or excessive inflammation can suppress osteogenesis, promote bone resorption, and disrupt the regenerative microenvironment. Anti-inflammatory therapy may therefore be beneficial in bone defects characterized by inflammatory dysregulation. However, because a physiological inflammatory response is required during the early phase of healing, the aim of local treatment should be to limit persistent or pathological inflammation rather than abolish inflammation altogether. Dose and duration of exposure are consequently important, as excessive or prolonged anti-inflammatory activity may interfere with normal repair (Huang et al., 2020; Hachemi et al., 2024).

Dexamethasone and aspirin are representative conventional anti-inflammatory agents whose local effects can be modified through biomaterial-based delivery. Sustained low-dose dexamethasone release from three-dimensional (3D)-printed polycaprolactone (PCL) scaffolds promoted macrophage phenotype switching and enhanced osteogenic differentiation of mesenchymal stromal cells, whereas dexamethasone delivered from sericin hydrogels improved repair in an *in vivo* bone-defect model (Majrashi et al., 2023; Fan et al., 2024). Aspirin incorporated into microsized tricalcium phosphate-coated macroporous gelatin microribbon scaffolds promoted reparative macrophage responses, reduced osteoclast activity, and enhanced bone regeneration in a dose-dependent manner. Similarly, an injectable hyaluronate hydrogel co-encapsulating acetylsalicylic acid and bone marrow mesenchymal stem cells (BMSCs) promoted osteogenic differentiation and M2 macrophage polarization, resulting in greater bone formation in rat calvarial defects (Su et al., 2023; Guo et al., 2024). The dose-dependent reduction in iNOS-positive macrophages associated with aspirin delivery from the microribbon scaffold is illustrated in Figure 2A.

**FIGURE 2:**
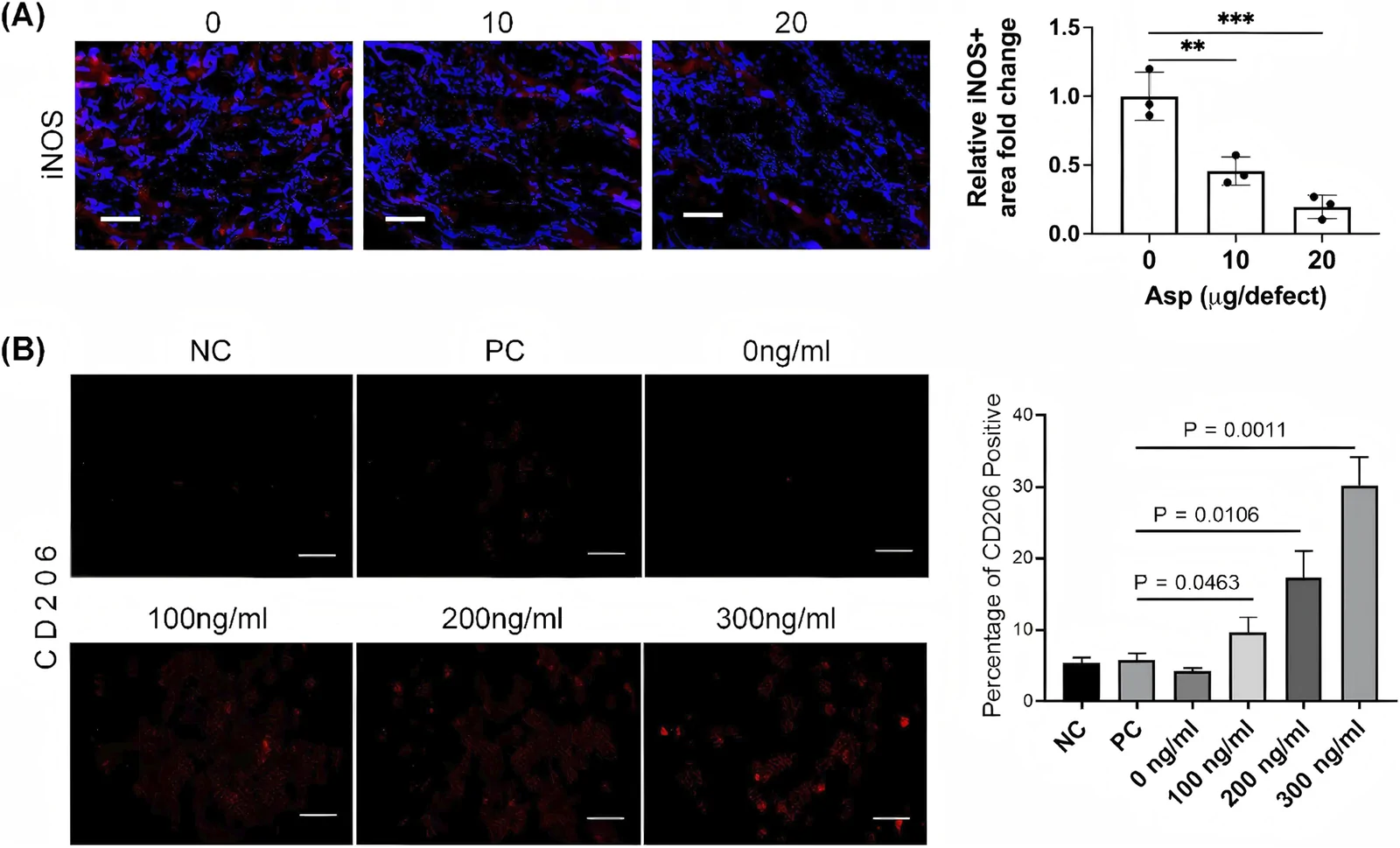
Representative immunomodulatory effects of locally delivered anti-inflammatory and immunoregulatory agents in bone regeneration. **(A)** Representative iNOS immunofluorescence images and corresponding quantification showing aspirin-associated reduction in iNOS-positive macrophages. Adapted from Figures 4A,B of the original study (Su et al., 2023). **(B)** Representative CD206 immunofluorescence images and corresponding quantification showing an increase in CD206-positive macrophages across the indicated conditions and interleukin-4 (IL-4) concentrations. Adapted from Figures 2A,C of the original study (Zhang et al., 2020). The two panels are derived from independent studies using different experimental models, analytical methods, and outcome measures; they are presented as representative examples and are not intended for direct quantitative comparison. Source panels were cropped, resized, rearranged, and/or relabeled for the composite layout under the respective Creative Commons Attribution (CC BY) licenses; no underlying experimental data were altered.

In defects where inflammation is accompanied by oxidative stress, simultaneous regulation of both processes may be advantageous. Dimethyl fumarate provides one example: a reactive oxygen species (ROS)-responsive hydrogel released the drug in response to the local oxidative environment, reducing pro-inflammatory mediators and intracellular ROS while enhancing osteogenic activity and accelerating cranial defect repair (Huang et al., 2025). This approach illustrates how the pathological microenvironment itself can be used to regulate local therapeutic exposure.

Natural small molecules with both anti-inflammatory and antioxidant properties offer another approach. Quercetin incorporated into a bioglass microsphere-containing injectable hydrogel alleviated oxidative stress and promoted osteogenic repair in periodontal bone defects (Zhu et al., 2024). Curcumin-loaded chitosan microspheres embedded in oxidized alginate–gelatin hydrogels prolonged curcumin release for approximately 3 weeks and enhanced osteogenic differentiation of human BMSCs *in vitro* (Amiryaghoubi et al., 2024). In another system, curcumin-loaded poly (lactic-co-glycolic acid) (PLGA) microspheres were incorporated into a copper-containing hydrogel, which reduced inflammation and oxidative stress, promoted reparative macrophage polarization, and further supported angiogenesis and osteogenesis through copper-ion release (Lu et al., 2025).

Taken together, these studies support local modulation of both inflammation and oxidative stress, but their findings are not directly comparable because the therapeutic agents, carrier compositions, and experimental models differ substantially. Moreover, in multifunctional constructs, regenerative effects cannot always be attributed to the anti-inflammatory compound alone. The available evidence therefore supports the general strategy of microenvironment-responsive local therapy rather than the superiority of any particular agent or carrier.

### 3.2 Immunoregulatory cytokines and chemokines

Immune dysregulation and insufficient recruitment of reparative cells represent distinct biological barriers to bone regeneration. Immunoregulatory cytokines can modify an unfavorable immune environment, whereas chemokines, as a specialized cytokine subgroup, can promote the migration and recruitment of reparative cells. Current local-delivery strategies include sustained administration of immunoregulatory cytokines such as interleukin-4 (IL-4) and interleukin-10 (IL-10), as well as chemokines that enhance the recruitment of reparative cell populations (Zhang et al., 2020; Wang et al., 2024).

IL-4 and IL-10 are among the most extensively studied immunoregulatory cytokines in bone regeneration. IL-4 primarily acts by modulating macrophage phenotype and improving the osteoimmune microenvironment. Sustained local IL-4 delivery from hydrogels, biodegradable microparticles, and PCL/nanohydroxyapatite biomimetic scaffolds has promoted reparative macrophage responses, reduced inflammation, and enhanced bone regeneration in bone-defect, periodontal, and critical-sized calvarial models (Zhang et al., 2020; Shehabeldin et al., 2024; Wang et al., 2026). The IL-4-associated increase in CD206-positive macrophages is illustrated in Figure 2B. IL-10 similarly limits persistent inflammation and supports restoration of immune homeostasis. Delivery through physically cross-linked DNA hydrogels and self-assembling peptide hydrogels has preserved IL-10 bioactivity, promoted reparative macrophage responses and mesenchymal-cell osteogenesis, reduced diabetic periodontal inflammation, and accelerated alveolar bone-defect repair (Li et al., 2022; Wang et al., 2023). For both cytokines, therapeutic performance depends not only on their intrinsic biological activity but also on maintaining an appropriate local concentration and duration of exposure.

Chemokines extend this strategy by promoting the migration and recruitment of reparative cells. Stromal cell-derived factor-1α (SDF-1α/CXCL12) is a representative example. Sustained SDF-1α delivery from 3D-printed PCL/bioactive-glass composite scaffolds and decellularized fish-scale scaffolds promoted BMSC migration and recruitment and improved bone repair in rabbit tibial and mouse calvarial defect models, respectively (Wang et al., 2024; Su et al., 2025). The SDF-1α-associated increase in BMSC migration observed in the 3D-printed composite scaffold system is illustrated in Figure 3A. This recruitment-oriented mechanism differs from the predominantly immunoregulatory roles of IL-4 and IL-10 by increasing the number of reparative cells available within the defect.

**FIGURE 3:**
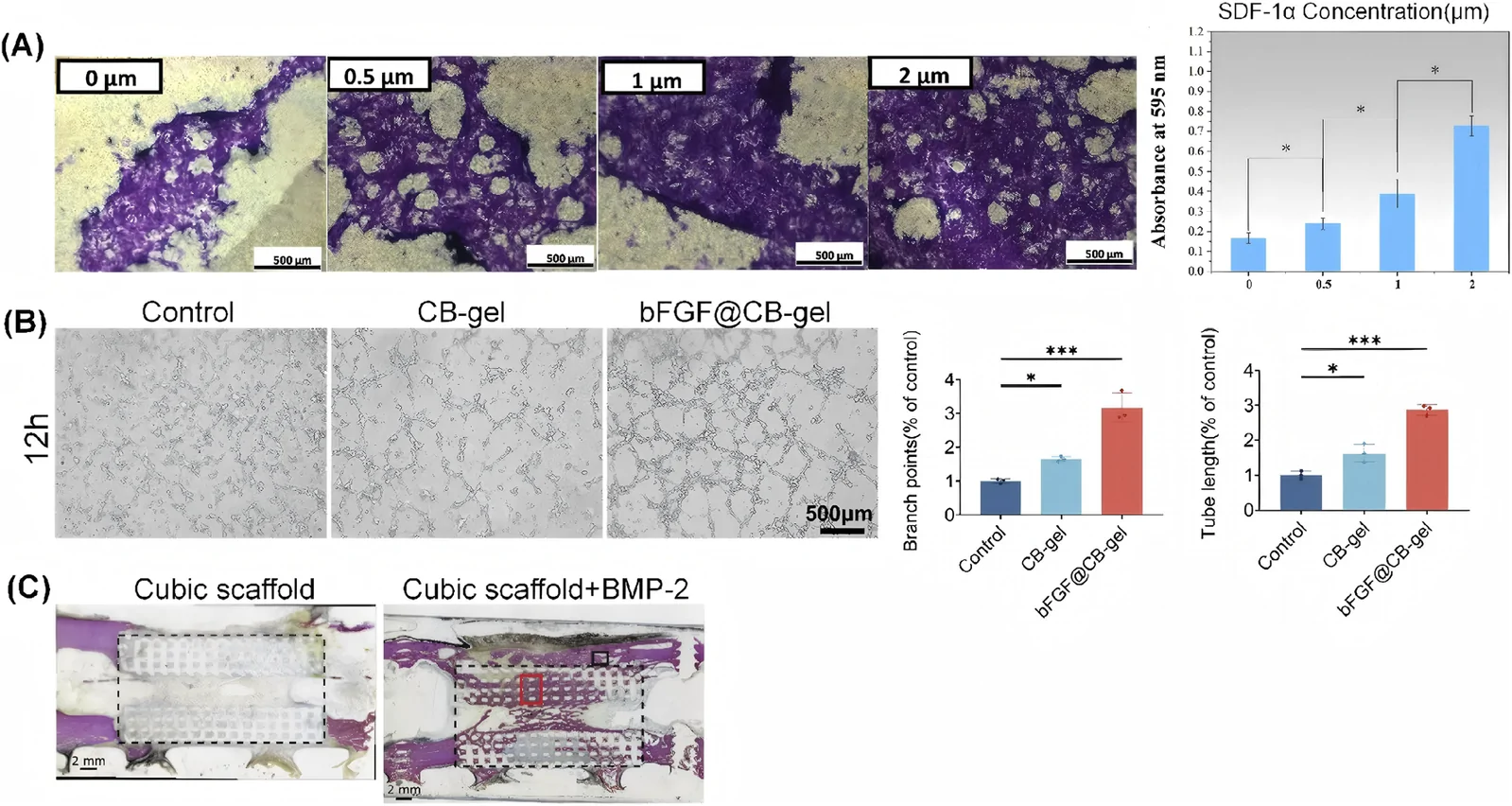
Representative cell-recruitment, angiogenic, and osteogenic effects of locally delivered therapeutic agents in bone regeneration. **(A)** Representative BMSC migration images and corresponding quantification following SDF-1α treatment. Adapted from Figures 1D,E of the original study (Wang et al., 2024). **(B)** Representative endothelial tube-formation images for the control, CB-gel, and bFGF@CB-gel groups, with corresponding quantification of branch points and tube length. Adapted from Figures 3G,H of the original study (Feng et al., 2026). **(C)** Representative histological sections comparing cubic scaffolds without and with bone morphogenetic protein-2 (BMP-2), illustrating BMP-2-associated bone formation in a sheep critical-size metatarsal defect. Adapted from Figure 8A,C of the original study (Garot et al., 2023). The three panels are derived from independent studies using different experimental models, analytical methods, and outcome measures; they are presented as representative examples and are not intended for direct quantitative comparison. Source panels were cropped, resized, rearranged, and/or relabeled for the composite layout under the respective Creative Commons Attribution (CC BY) licenses; no underlying experimental data were altered.

Interleukin-8 (IL-8/CXCL8) has also been explored as a recruitment-oriented chemokine. A composite scaffold comprising IL-8, transferable decellularized matrix, and PLGA promoted the recruitment of BMSCs and macrophages through C-X-C motif chemokine receptor 2 signaling and increased new bone mineralization and maturation in a rat femoral defect model (Zhao et al., 2025). Although evidence for IL-8-based delivery in bone regeneration remains limited, this study suggests that chemokine delivery can be used not only to modify the behavior of cells already present within the defect but also to recruit additional reparative populations.

Within the broader cytokine family, immunoregulatory cytokines and chemokines therefore address distinct but complementary therapeutic needs: regulation of an unfavorable immune environment and recruitment of cells required for regeneration. Selection of an appropriate strategy should depend on the dominant biological deficit, while local concentration, exposure duration, and release timing remain important determinants of therapeutic performance.

### 3.3 Angiogenic growth factors

Adequate vascular reconstruction is essential for bone-defect repair because newly forming tissue requires sustained perfusion, nutrient supply, and vascular support for cellular recruitment and function. Local delivery of angiogenic growth factors can enhance endothelial-cell recruitment, migration, vascular sprouting, and vessel organization. Vascular endothelial growth factor (VEGF), fibroblast growth factors (FGFs), and platelet-derived growth factor-BB (PDGF-BB) are among the most widely investigated molecules, although their vascular functions and optimal exposure profiles differ (Street et al., 2002; Jang and Yoon, 2024).

VEGF is a central regulator of angiogenesis and remains one of the most extensively studied factors for local vascular stimulation. A Laponite-modified gelatin methacryloyl (GelMA) hydrogel provided controlled vascular endothelial growth factor A (VEGF-A) delivery, promoted endothelial progenitor-cell recruitment and neovascularization, and enhanced cranial bone regeneration (Mi et al., 2024). Polydopamine-functionalized strontium alginate/hydroxyapatite microhydrogels similarly combined sustained strontium-ion release with controlled VEGF delivery, resulting in enhanced angiogenesis and bone formation (Chen S. et al., 2024). These findings indicate that the regenerative value of VEGF depends not simply on its presence within a construct but also on maintaining effective local exposure.

Fibroblast growth factors provide another route to vascular stimulation. Fibroblast growth factor 1 (FGF-1; acidic FGF) incorporated into an MXene-based photoresponsive hydrogel underwent near-infrared-responsive release and promoted rapid angiogenesis and reconstruction of the osteovascular network during bone repair (Wu et al., 2024). Fibroblast growth factor 2 (FGF-2; basic FGF) promoted microvascular endothelial-cell migration and angiogenesis through fibroblast growth factor receptor-mediated pro-angiogenic signaling, while sustained local delivery increased vascular density in a critical-sized bone-defect model (Feng et al., 2026). Representative FGF-2-enhanced endothelial tube formation, together with quantitative measures of branch points and tube length, is shown in Figure 3B. Together, these studies show that FGF delivery can combine direct pro-angiogenic activity with material-based control of local exposure.

PDGF-BB contributes to the establishment and organization of a functional vascular microenvironment. In radiation-induced bone damage, disruption of hypoxia-inducible factor-1α (HIF-1α)/PDGF-BB/PDGF receptor β signaling impaired the association between type H endothelial cells and PDGF receptor β-positive perivascular cells, contributing to vascular injury and bone loss (Li J. et al., 2023). PDGF-BB has also been used as an early angiogenic signal in a sequential delivery system in which subsequent bone morphogenetic protein-2 (BMP-2) release supported vascularized bone regeneration (Niu et al., 2024). These findings support a role for PDGF-BB not only in promoting vascular responses but also in coordinating vascular-cell interactions that favor subsequent bone formation.

Placental growth factor (PlGF) further illustrates the importance of local exposure. PlGF-loaded alginate microparticles incorporated into collagen/hydroxyapatite scaffolds were designed to provide a relatively high early exposure favoring angiogenesis, followed by lower sustained exposure supporting osteogenesis; this delivery pattern enhanced repair of critical-sized calvarial defects (Sheehy et al., 2021). Thus, the effectiveness of an angiogenic factor may depend as much on the magnitude and duration of local exposure as on the identity of the factor itself.

Angiogenic growth-factor delivery should therefore be designed to establish a functional vascular network rather than simply to maximize vessel formation. Depending on the biological deficit, this may require endothelial recruitment and sprouting, stabilization and organization of vascular-cell interactions, or coordination between angiogenesis and subsequent osteogenesis. Factor selection and delivery design should consequently be considered together, with particular attention to local dose, timing, and duration of exposure.

### 3.4 Osteogenic growth factors

Osteogenic growth factors directly support bone formation by promoting osteogenic differentiation, osteoblast activity, and matrix mineralization. Among these molecules, bone morphogenetic proteins (BMPs) remain the most established, particularly BMP-2. The central challenge in local delivery is therefore not simply to provide a potent osteogenic stimulus, but to maintain sufficient activity within the defect while limiting excessive exposure and associated adverse effects (Ball et al., 2023; Chernysheva et al., 2025).

Bone morphogenetic protein-2 (BMP-2) is the principal growth factor used for local osteogenic stimulation. Its strong osteoinductive activity is particularly relevant to critical-sized defects with limited intrinsic regenerative capacity, although high local exposure can be detrimental. Low-dose BMP-2 immobilized on 3D-printed polylactic acid (PLA) scaffolds through a polyelectrolyte film achieved substantial regeneration in a critical-sized sheep metatarsal defect without obvious adverse effects (Garot et al., 2023). Representative histological evidence of BMP-2-associated bone formation in the cubic scaffold system is shown in Figure 3C. Sustained delivery has also been pursued using a carboxymethyl chitosan/polyethylene glycol (PEG)/heparin sulfate hydrogel, which promoted osteogenic differentiation and cranial bone regeneration (Zhou T. et al., 2024). In another approach, bilayer oxidized sodium alginate–carboxymethyl chitosan microspheres prolonged BMP-2 release, enhanced osteogenic differentiation of rat bone marrow mesenchymal stem cells, and supported ectopic osteogenesis when combined with calcium phosphate cement (Yuan et al., 2025). These studies collectively emphasize control of local BMP-2 exposure rather than simple dose escalation.

BMP-7 provides an alternative osteoinductive signal, although recent evidence for biomaterial-based local delivery is less extensive than that for BMP-2. A decellularized muscle scaffold incorporating BMP-7 and silica calcium phosphate cement and seeded with human mesenchymal stromal cells enhanced new bone formation and maturation in a critical-sized calvarial defect model compared with the corresponding construct without BMP-7 (Alfotawi, 2024). The available evidence therefore supports the osteogenic potential of BMP-7 while also indicating that its contemporary local-delivery literature remains comparatively limited.

Insulin-like growth factor-1 (IGF-1) has a different biological profile, supporting osteoblast proliferation, differentiation, and matrix synthesis rather than providing the strong osteoinductive stimulus characteristic of BMPs. A PLA/PEG/bredigite 3D-printed scaffold coated with chitosan/PEG nanofibers and co-delivering vancomycin and IGF-1 provided sustained release for 28 days and promoted repair in a rat calvarial defect model (Salehi et al., 2025). Because this construct combined IGF-1 with vancomycin and several bioactive scaffold components, however, the regenerative effect cannot be attributed to IGF-1 alone. This illustrates an important interpretive limitation of multifunctional delivery systems when the contribution of individual therapeutic components is not directly separated.

NEL-like protein-1 (NELL-1) represents another osteogenic signal with potential for local delivery. Earlier work demonstrated that adenoviral NELL-1 delivered using a demineralized bone matrix carrier enhanced spinal fusion in rats (Lu et al., 2007). More recently, a 3D-bioprinted microfiber-in-calcium phosphate cement platform enabled spatiotemporal NELL-1 delivery and enhanced osteogenesis together with vascularization and innervation in rat cranial defects (Zhu et al., 2026). These findings suggest that NELL-1 may support broader regenerative interactions beyond osteogenesis alone, although the evidence base remains considerably smaller than that for BMP-2.

Osteogenic growth-factor delivery should therefore be tailored to the degree and nature of impaired bone-forming capacity. For BMP-2 in particular, improving local retention and controlling effective exposure may be more important than increasing the administered dose. Alternative factors such as BMP-7, IGF-1, and NELL-1 may provide distinct forms of osteogenic support, but their value should be interpreted in light of the strength of the available evidence and the contribution of other components within complex delivery systems. Representative biological effects of the locally delivered therapeutic agents discussed above are summarized in Figures 2, 3.

### 3.5 Combined therapeutic strategies

Complex bone defects frequently involve several concurrent biological limitations, including inflammatory dysregulation, inadequate vascularization, insufficient recruitment of reparative cells, and reduced osteogenic capacity. In such settings, a single therapeutic agent may not address all of the processes limiting regeneration. Combination therapy can therefore be used to target distinct but complementary deficits, provided that each component has a defined biological role. Common strategies include angiogenic–osteogenic, immunomodulatory–osteogenic, and cell-recruitment–osteogenic combinations. The value of a multi-agent system depends not only on the biological functions of its components but also on their doses, relative timing, duration of exposure, and spatial organization.

VEGF and BMP-2 represent a widely investigated angiogenic–osteogenic pairing, combining vascular stimulation with direct osteoinductive activity. Dual-crosslinked alginate/gelatin methacryloyl (GelMA) microspheres carrying VEGF and BMP-2 promoted angiogenesis and bone repair, while the Cu^2+^-crosslinked component also contributed antibacterial activity (Dai et al., 2024). A bilayer 3D-bioprinted GelMA scaffold incorporating BMP-2 and VEGF likewise enhanced vascularized bone regeneration in a calvarial defect model (Alarcin et al., 2025). This combination is biologically plausible because vascular reconstruction and osteogenesis are closely coupled during bone repair, although the benefit of combining both factors still depends on their dose and delivery context.

Immunomodulatory–osteogenic combinations may be useful when persistent inflammation coexists with impaired bone formation. A nanoparticle/hydrogel hybrid system combining IL-10 with icariin used IL-10 to improve the local immune environment while icariin promoted osteogenic differentiation of bone marrow mesenchymal stem cells (BMSCs). Their combined delivery enhanced bone regeneration in a rat critical-sized cranial defect model (Li X. et al., 2024). This approach illustrates how agents acting on different components of the regenerative microenvironment can be incorporated within the same therapeutic system.

Recruitment signals can likewise be paired with osteogenic factors when an insufficient supply of reparative cells limits healing. In a scaffold containing stromal cell-derived factor-1α (SDF-1α) and BMP-2, SDF-1α promoted BMSC recruitment, whereas BMP-2 supported subsequent osteogenic differentiation and bone formation (Wei et al., 2023). A related combination of SDF-1α and insulin-like growth factor-1 (IGF-1) enhanced BMSC migration, osteogenic marker expression, and matrix mineralization and improved regeneration in a rat critical-sized calvarial defect model (Zhong et al., 2024). These examples link cell recruitment with subsequent osteogenic support rather than relying on either process alone.

Other combinations seek to reproduce the coordinated signaling environment of bone healing. An injectable poly (organophosphazene) hydrogel containing transforming growth factor-β1 (TGF-β1) and BMP-2 was designed to provide distinct release behavior for the two factors. The construct increased early angiogenic and stemness-related markers and was progressively replaced by newly formed bone *in vivo* (Kim et al., 2023). Such systems highlight the potential importance of coordinating not only therapeutic functions but also their temporal exposure.

Functional complementarity, however, does not guarantee therapeutic synergy. In a composite injury model combining a femoral segmental bone defect with volumetric muscle loss, simultaneous or sequential delivery of VEGF and BMP-2 did not provide a clear regenerative advantage over BMP-2 alone, and the mechanical properties of the regenerated bone remained substantially below those of intact bone (Subbiah et al., 2020). Thus, the rationale for combining agents should be supported by direct comparison with simpler treatment strategies rather than inferred solely from their complementary biological functions.

Combination therapy is most justified when multiple well-defined barriers to repair must be addressed within the same defect. Increasing the number of therapeutic agents should therefore not be treated as an advantage in itself. Overall, the evidence base remains uneven across therapeutic categories: BMP-2 has been investigated extensively, whereas several cytokines, chemokines, alternative osteogenic factors, and emerging multi-agent strategies are supported by a smaller and more heterogeneous body of evidence and should therefore be interpreted more cautiously. How these exposure patterns can be controlled through biomaterial design is discussed in Section 5.

## 4 Biomaterial scaffolds for therapeutic delivery in bone regeneration

The effectiveness of local therapeutic delivery depends not only on the selected agent but also on whether the carrier is compatible with both the cargo and the defect environment. Scaffold selection should account for defect geometry, cargo stability and retention, implantation requirements, and the need for structural support. Hydrogels, porous solid scaffolds, fibrous scaffolds, and composite systems offer distinct combinations of these properties and are therefore suited to different regenerative and delivery requirements (Szwed-Georgiou et al., 2023; Ehlen et al., 2024). Their cross-platform trade-offs in structural support, defect conformity, therapeutic-agent loading and protection, release control, and major limitations are compared in Table 2.

**TABLE 2:** Comparison of major biomaterial scaffold platforms for local therapeutic delivery in bone regeneration.

Scaffold platform	Structural support and defect conformity	Therapeutic-agent loading and protection	Release-control features	Potential use and limitations	References
Hydrogel scaffolds	Limited bulk mechanical support; high conformity to irregular defects; injectable/in situ-forming formulations can adapt closely to defect geometry	Mild incorporation of drugs, proteins, cytokines, and growth factors; hydrated matrices can help preserve labile cargo	Tunable through crosslinking, degradation, affinity interactions, and stimulus responsiveness	Irregular defects and minimally invasive delivery; limited load-bearing support	(Cheon et al., 2024; Cohen et al., 2025)
Porous solid scaffolds	Prolonged space maintenance and structural support while permitting cell infiltration, vascular ingrowth, and new bone formation	Therapeutics can be incorporated during fabrication or immobilized by adsorption, coatings, or functionalization	Pore architecture, degradation, surface binding, and spatial cargo localization	Defects requiring structural support; lower conformity to irregular defects	(Takase et al., 2024; Qin et al., 2025)
Fibrous scaffolds	Provide a fiber-based interface for cell attachment and partly mimic the native fibrous environment	Therapeutics can be incorporated within fibers or immobilized on their surfaces; layered or core–shell structures enable spatially organized delivery	Fiber composition, layering, core–shell design, and degradation	Biomimetic interfaces and local delivery; limited bulk structural support	(Sadeghi-Ghadikolaei et al., 2024; Sriram et al., 2024)
Composite scaffolds	Combine structural support, defect filling, and therapeutic delivery through complementary components	Therapeutics can be distributed among distinct material compartments or delivery units	Compartmentalized, independent, or sequential release from distinct components	Integration of structural and delivery functions; increased fabrication complexity	(Spiller and Vunjak-Novakovic, 2015; Kitahara et al., 2024; Zhu et al., 2025)

### 4.1 Hydrogel scaffolds

Hydrogels are particularly useful when conformal filling of irregular defects, minimally invasive implantation, and mild incorporation of bioactive agents are priorities. Their highly hydrated polymer networks can provide a favorable environment for small-molecule drugs and protein therapeutics, including cytokines and growth factors, while injectable or in situ-forming formulations can adapt closely to complex defect geometries (Cheon et al., 2024; Cohen et al., 2025). An injectable hyaluronic acid-based hydrogel, for example, underwent post-injection self-curing and progressively increased in stiffness, supporting bone regeneration in a rat femoral defect model (Cheon et al., 2024). The injectability and microstructural characteristics of this HAps hydrogel are illustrated in Figure 4A. A rapidly crosslinking polyethylene glycol (PEG) hydrogel could similarly be delivered in liquid form, conform to cranial defects, and polymerize at body temperature while locally delivering recombinant human bone morphogenetic protein-2 (rhBMP-2) (Cohen et al., 2025). These properties make hydrogels especially attractive when local retention and defect conformity are more important than prolonged load-bearing support.

**FIGURE 4:**
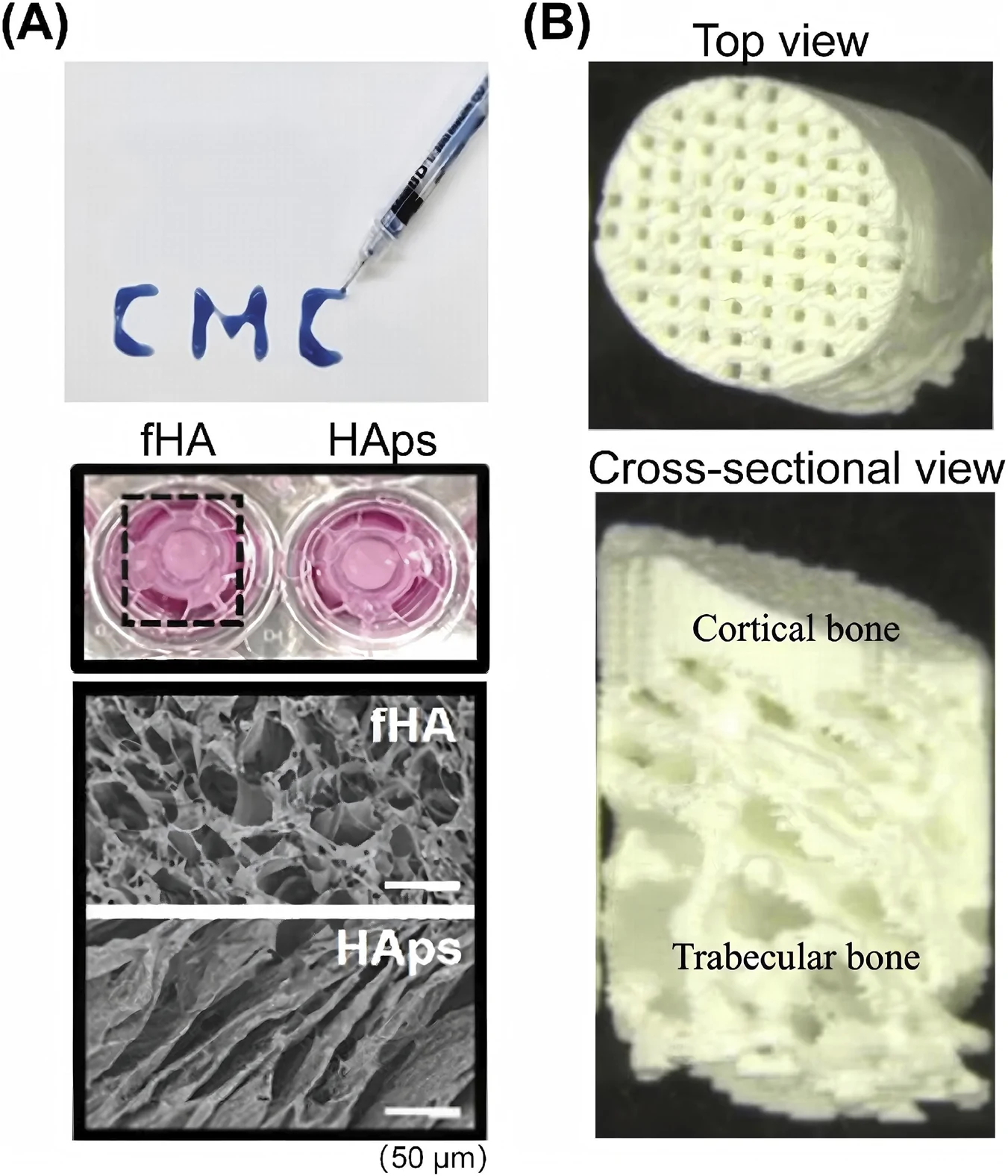
Representative architectures and structural characteristics of hydrogel and porous solid scaffolds for bone regeneration. **(A)** Representative images showing the injectability and microstructure of an HAps hydrogel. Adapted from Figures 1H, 3A of the original study (Cheon et al., 2024). **(B)** Top and cross-sectional views of a three-dimensional (3D)-printed calcium phosphate scaffold designed to emulate cortical and trabecular bone microarchitecture. Adapted from Figure 1 of the original study (Takase et al., 2024). The two panels are derived from independent studies using different material systems and imaging or analytical methods; they are presented as representative structural examples and are not intended for direct quantitative comparison. Source panels were cropped, resized, rearranged, and/or relabeled for the composite layout under the respective Creative Commons Attribution (CC BY) licenses; no underlying experimental data were altered. fHA, free hyaluronic acid; HAps, hyaluronic acid modified with phenylboronic acid and spermidine (HA-PBA-SM).

The versatility of hydrogels also allows them to accommodate therapeutics directed at different biological barriers. A linagliptin-loaded dynamic hydrogel modulated the inflammatory microenvironment in diabetic bone defects while improving vascularization and bone regeneration (Yang P. et al., 2025). A VEGF-containing composite hydrogel promoted angiogenesis and bone repair in critical-sized cranial defects (Huang et al., 2024), whereas the PEG hydrogel described above improved rhBMP-2 localization and reduced off-target bone formation compared with a collagen-sponge carrier. Hydrogels can therefore support immunomodulatory, angiogenic, and osteogenic interventions rather than being restricted to a single class of therapeutic cargo.

Hydrogel performance, however, is determined by more than cargo selection. Matrix mechanics, cell-adhesive properties, degradation, and biochemical functionalization can themselves influence the regenerative response. In engineered PEG hydrogels delivering BMP-2, variations in matrix stiffness, Arg-Gly-Asp-mediated cell adhesion, and degradation altered cell spreading and migration and ultimately affected bone regeneration in rat calvarial defects (Vallmajo-Martin et al., 2024). Macroporous hydrogels with tunable mechanical properties and degradation provided space for cell proliferation while maintaining mechanical cues favorable for osteogenic differentiation, with regenerative efficacy demonstrated in rabbit and porcine models (Xue et al., 2025). Biochemical functionalization can further exploit endogenous regenerative signals; for example, a functionalized hyaluronic acid methacryloyl hydrogel captured endogenous BMP-2 and promoted cranial bone regeneration (Yin et al., 2025). Hydrogels should therefore be regarded not simply as passive depots but as material environments that can influence cell behavior and tissue repair.

Their principal limitation is comparatively weak long-term structural and mechanical support. Hydrogels are consequently best suited to applications in which defect conformity, minimally invasive implantation, and localized therapeutic delivery are primary requirements. Large or load-bearing defects requiring prolonged space maintenance may instead benefit from porous solid scaffolds, whereas composite systems can combine the structural support of a solid framework with the delivery flexibility of a hydrogel. Improving mechanical performance without sacrificing injectability or biological compatibility remains an important design challenge for hydrogel-based bone regeneration.

### 4.2 Porous solid scaffolds

Porous solid scaffolds are particularly suited to bone defects that require sustained space maintenance and structural support. Their interconnected pore networks provide pathways for cell infiltration, nutrient transport, vascular ingrowth, and new bone formation, while the solid framework helps preserve defect geometry during regeneration. Scaffold performance depends not only on overall porosity but also on pore size, interconnectivity, and spatial organization, all of which influence tissue penetration and mass transport (Takase et al., 2024; Qin et al., 2025).

3D printing provides a means of controlling these architectural features with high spatial precision. Hydroxyapatite-containing PLGA scaffolds with directional microchannels have been developed to facilitate cell migration and nutrient transport (Cheng et al., 2024). Calcium phosphate scaffolds designed to reproduce cortical- and trabecular-like microstructures supported progressive bone ingrowth toward the scaffold interior in rabbit femoral defects (Takase et al., 2024). Top and cross-sectional views of the cortical- and trabecular-like scaffold architecture are shown in Figure 4B. Similarly, PCL-based scaffolds incorporating silk fibroin and β-tricalcium phosphate with different pore scales in the inner and outer regions improved hydrophilicity, tissue ingrowth, and femoral defect repair (Qin et al., 2025). These studies indicate that the spatial organization of pores can be as important as overall porosity in determining scaffold performance.

Porous solid scaffolds can also function as local carriers for therapeutic agents. Cargo can be incorporated during fabrication or immobilized after fabrication through adsorption, coatings, or surface functionalization. Ketoprofen incorporated directly into 3D-printed PLGA scaffolds showed release behavior that varied with infill density, wall thickness, and scaffold geometry (Annaji et al., 2024). Two-dimensional hetero-nanostructures have also been used to increase IL-4 loading on 3D-printed scaffolds and promote a pro-healing immune microenvironment (Liu et al., 2023). In another approach, a Laponite nanoclay coating was used to bind BMP-2 to 3D-printed scaffolds, improving local factor retention and enhancing bone formation in an ovine femoral defect model (Marshall et al., 2024). These examples demonstrate how a structurally supportive scaffold can simultaneously serve as a platform for localized therapeutic delivery.

Importantly, some porous scaffolds can modify the regenerative microenvironment even without externally loaded drugs, cytokines, or growth factors. A biodegradable 3D-printed zinc-based porous scaffold integrating alloy composition, surface microstructure, and macroscopic pore architecture promoted an anti-inflammatory macrophage response, collagen deposition, and new bone formation (Li S. et al., 2024). Likewise, a 3D-printed poly (L-lactic acid)-based scaffold containing β-tricalcium phosphate and calcium sulfate promoted reparative macrophage polarization, increased IL-10, VEGF, and BMP-2 secretion, and enhanced regeneration in rabbit segmental bone defects (Cen et al., 2025). These findings reinforce the view that scaffold composition and architecture can themselves contribute to osteoimmune and regenerative regulation.

The principal advantage of porous solid scaffolds is their ability to combine prolonged space maintenance with tissue ingrowth and localized delivery. Their design, however, involves an inherent trade-off between porosity and mechanical stability, and preformed solid constructs generally conform less readily than injectable hydrogels to highly irregular defects. When both structural support and adaptable therapeutic delivery are required, combining a porous framework with hydrogel or other delivery components may offer a more suitable solution.

### 4.3 Fibrous scaffolds

Fibrous scaffolds provide a microscale interface for cell attachment, spreading, and tissue organization. Because native bone contains a collagen-rich extracellular matrix, fibrous architectures can reproduce some features of the natural environment surrounding bone cells while also providing surfaces for local therapeutic loading. Electrospinning is widely used to fabricate such scaffolds because it allows control over fiber morphology and organization. Conventional electrospun membranes, however, are often thin and densely packed, which can restrict cell penetration and vascular ingrowth into deeper regions of the construct (Sadeghi-Ghadikolaei et al., 2024; Sriram et al., 2024).

Several design strategies have been developed to overcome these limitations. A 3D scaffold assembled from electrospun chitosan/polyvinyl alcohol fibers and mineralized with brushite contained both small interfibrous pores and larger 3D pores, thereby improving cellular infiltration (Sadeghi-Ghadikolaei et al., 2024). Mineralized aligned polyhydroxybutyrate-based fibers combined with tubular structures were designed to reproduce features of mineralized collagen fibers and osteon-like organization (Sriram et al., 2024). Coaxial electrospinning has also been used to produce polyglycolic acid/silk fibroin core–shell fibers with complementary mechanical and degradation properties, promoting vascularization and bone formation in rat calvarial defects (Zhang Y. et al., 2024). Together, these approaches address limitations of conventional electrospun membranes by introducing greater structural depth, biomimetic organization, and control over degradation.

Fibrous scaffolds can also act as local therapeutic-delivery platforms. Bioactive agents may be incorporated within fibers during fabrication or immobilized on the fiber surface. Melatonin-encapsulated silk fibroin electrospun nanofibers provide an example of direct therapeutic incorporation within a fibrous matrix (Deng et al., 2024). The gross appearance and multiscale fibrous morphology of these melatonin-loaded silk fibroin nanofibers are shown in Figure 5A. A layered electrospun scaffold separately incorporated aspirin and osteogenic growth peptide, thereby combining modulation of the inflammatory environment with support for periodontal bone regeneration (Mu et al., 2025). Coaxially electrospun fibers containing interleukin-4 (IL-4) promoted a favorable immune environment and calvarial repair (Jiang et al., 2024), whereas polydopamine-modified nanofibers were used to immobilize bone morphogenetic protein-2 (BMP-2), enhancing osteogenic differentiation and bone-defect healing (Chen Z. et al., 2024). These examples demonstrate that fibrous systems can accommodate small molecules, cytokines, and osteogenic factors through different loading strategies. More detailed control of release rate, duration, and sequence is discussed in Section 5.

**FIGURE 5:**
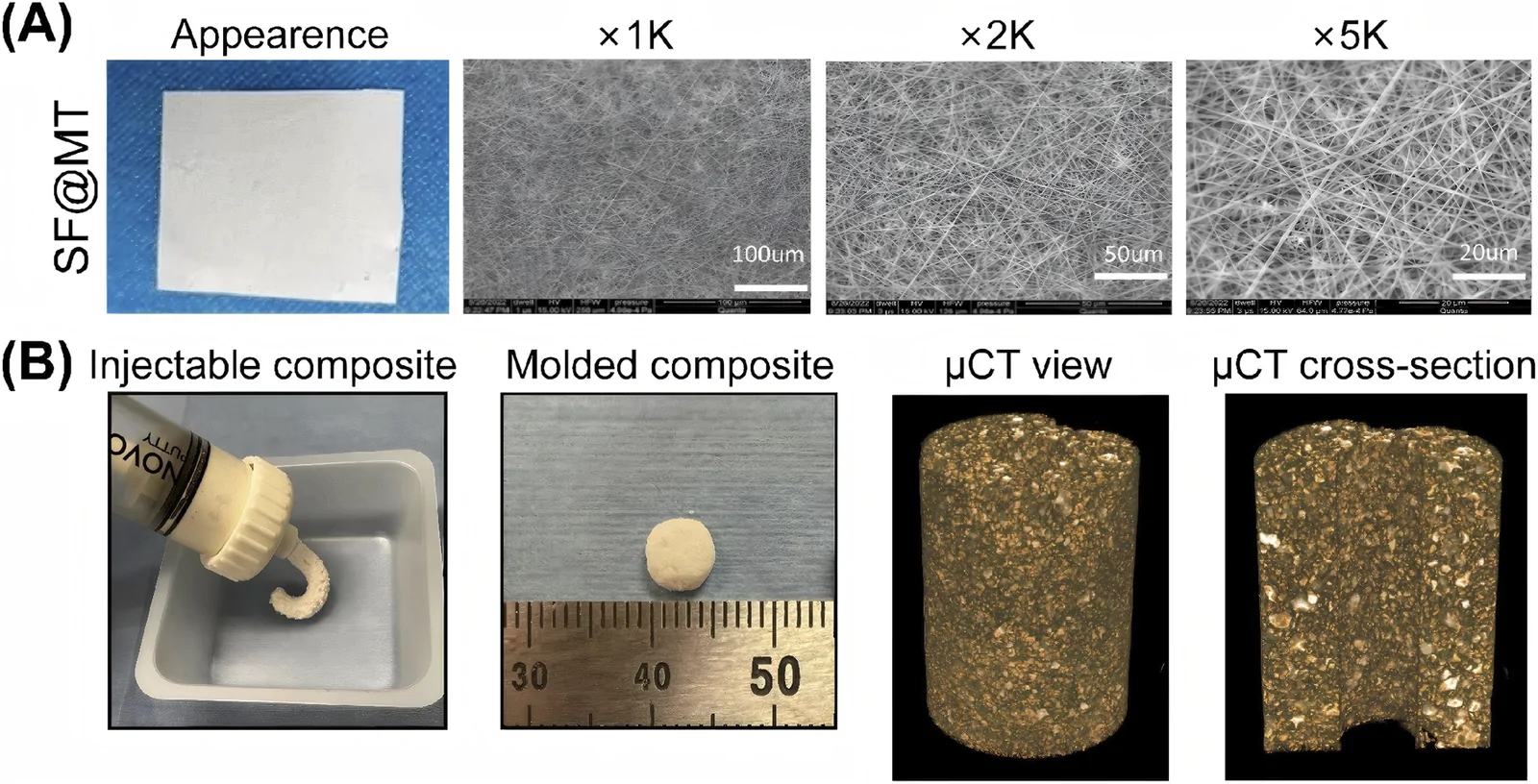
Representative morphology and structural characteristics of fibrous and composite scaffolds for bone regeneration. **(A)** Gross appearance and scanning electron microscopy (SEM) morphology of melatonin-encapsulated silk fibroin electrospun nanofibers (SF@MT). Adapted from Figure 2A of the original study (Deng et al., 2024). **(B)** Hydroxyapatite (HA) granule/β-tricalcium phosphate (β-TCP) microsphere/poloxamer 407-based hydrogel composite showing injectability, moldability, and internal ceramic distribution by micro-computed tomography (μCT). Adapted and rearranged from Figures 1A,B of the original study (Kitahara et al., 2024). The two panels are derived from independent studies using different material systems and imaging or analytical methods; they are presented as representative structural examples and are not intended for direct quantitative comparison. Source panels were cropped, resized, rearranged, and/or relabeled for the composite layout under the respective Creative Commons Attribution (CC BY) licenses; no underlying experimental data were altered.

Fibrous scaffolds are particularly useful when a biomimetic cellular interface and localized therapeutic delivery are important design requirements. Their limitations become more apparent in large defects: densely packed electrospun structures may restrict deep cellular and vascular infiltration, while fibrous constructs generally provide less bulk structural support than porous solid scaffolds. Integration with porous frameworks, hydrogels, or other material components may therefore be preferable when both a fibrous interface and substantial structural support are required.

### 4.4 Composite scaffolds

Composite scaffolds combine materials or delivery units with complementary properties when the structural, geometric, and therapeutic requirements of a bone defect cannot be adequately met by a single platform. A rigid or porous framework can provide space maintenance and mechanical support, a hydrogel can conform to internal voids and encapsulate bioactive agents, and microspheres or microparticles can serve as discrete loading units within the larger construct. The purpose of such combinations should therefore be functional integration rather than material complexity itself (Kitahara et al., 2024; Zhu et al., 2025).

Several recent systems illustrate this division of roles. A deferoxamine-loaded gelatin methacryloyl (GelMA) hydrogel was applied to a 3D-printed poly (glycolide-co-caprolactone)/hydroxyapatite porous scaffold, retaining the solid framework while adding angiogenic, antioxidative, and immunoregulatory functions (Zhu et al., 2025). Similarly, a zoledronic acid-loaded GelMA/polyacrylamide hydrogel was incorporated into a 3D-printed porous titanium scaffold, allowing the rigid framework and drug-containing hydrogel to provide complementary structural and therapeutic functions (Chu et al., 2024). A more compartmentalized design combined a 3D-printed PCL/nanohydroxyapatite framework with an alginate/gelatin hydrogel containing dexamethasone-loaded PCL microparticles, assigning structural support, defect filling, and drug delivery to different components of the system (Noory et al., 2025).

Microsphere–hydrogel composites offer further flexibility by allowing therapeutic agents with different properties or functions to be carried in separate compartments. GelMA/heparin methacrylate/nanohydroxyapatite microspheres bearing PDGF-BB were incorporated into a simvastatin-containing GelMA hydrogel, enabling the growth factor and small-molecule drug to be delivered from different components of the same construct (Zhou J. et al., 2024). In another system, β-tricalcium phosphate (β-TCP) microspheres were mixed with a poloxamer 407-based hydrogel and combined with hydroxyapatite (HA) granules to form an injectable and moldable composite containing rhBMP-2, which promoted bone healing in a rat femoral nonunion model (Kitahara et al., 2024). Across these systems, the common advantage is functional compartmentalization rather than any single material combination. However, because the cargos, scaffold architectures, and defect models differ and direct comparisons with simpler carriers are uncommon, the therapeutic benefit attributable specifically to increased material complexity remains uncertain. The injectable and moldable configuration of this composite, together with the internal distribution of its ceramic components, is illustrated in Figure 5B.

Composite platforms can also accommodate multiple therapeutic signals within a single construct. A 3D-printed microsphere–hydrogel scaffold incorporated nerve growth factor-mimetic and BMP-2-mimetic peptides to support reinnervation and osteogenesis (Zhang X. et al., 2025), whereas a 3D-printed GelMA scaffold containing alginate microspheres was used to deliver VEGF and BMP-2 within the same system (Zhou et al., 2025). Such designs provide opportunities to separate the loading or release of therapeutics with different physicochemical and biological requirements, although increasing the number of functional components also increases fabrication and analytical complexity.

Composite scaffolds are most useful when different material components solve distinct problems that cannot be addressed effectively by a simpler system. Their modularity can integrate structural support, defect conformity, and therapeutic delivery, but additional materials and fabrication steps may also complicate reproducibility, quality control, sterilization, and eventual clinical translation (Spiller and Vunjak-Novakovic, 2015; Herczeg and Song, 2022). Composite design should therefore be justified by a defined functional requirement rather than by complexity alone. Representative architectures and structural features of the four major scaffold platforms discussed above are shown in Figures 4, 5.

## 5 Controlled release strategies

The therapeutic performance of a locally delivered agent depends not only on its identity and dose but also on its release profile after incorporation into a biomaterial carrier. Release behavior is influenced by material architecture and degradation, interactions between the carrier and therapeutic cargo, and the location of the cargo within the delivery system. By modifying these features, biomaterials can regulate release rate, duration, sequence, and spatial distribution. Release can also be coupled to pathological cues within the defect or to externally applied stimuli. The relevant design goal is not maximal prolongation of release, but an exposure profile that is appropriate for the biological action of the therapeutic agent and the stage of repair (Szwed-Georgiou et al., 2023; Takematsu et al., 2023).

### 5.1 Control of release rate and duration

Release rate describes how rapidly a therapeutic agent is liberated from its carrier, whereas release duration refers to the period over which release continues. These parameters are related but should not be treated as interchangeable: slower release does not necessarily imply a therapeutically appropriate duration, and prolonged release is not inherently beneficial. Their optimal values depend on the biological activity of the therapeutic agent and the temporal requirements of bone repair. In biomaterial-based systems, release kinetics can be regulated through scaffold structure, carrier degradation, cargo distribution, and interactions that retain the therapeutic agent within the material.

Material structure can directly influence the rate at which cargo escapes from a carrier. Baek et al. incorporated calcium sulfate dihydrate into BMP-2-loaded hydroxyapatite microspheres. Dissolution of the calcium sulfate increased pore size and porosity and accelerated BMP-2 release by approximately 2.4-fold compared with microspheres without calcium sulfate dihydrate (Baek et al., 2018). This example shows how changes in internal material architecture can alter diffusional access and thereby modify release kinetics.

Carrier degradation provides another means of controlling release over time. Microspheres based on biodegradable poly (3-hydroxybutyrate-co-3-hydroxyvalerate-co-3-hydroxyhexanoate) (PBVHx) were used for BMP-2 delivery, with BMP-2 release largely following degradation of the polymeric carrier. Approximately 62.8% of the loaded BMP-2 was released within 4 weeks, with release continuing thereafter (Zhang et al., 2025b). In such degradation-coupled systems, the degradation behavior of the carrier becomes an important determinant of the release profile.

The location of therapeutic cargo within a scaffold can also alter release behavior. Qiu et al. compared direct adsorption of BMP-2 onto a silk fibroin/chitosan/mesoporous hydroxyapatite scaffold with preloading BMP-2 into the mesoporous nanoparticles before scaffold fabrication. Preloading reduced the initial burst and produced a more sustained release profile (Qiu et al., 2020). Thus, release can be modified not only by changing the bulk material but also by controlling where the therapeutic agent is positioned within the construct.

Affinity-based retention offers a further strategy for limiting rapid cargo loss. Jeon et al. immobilized heparin within photocrosslinked alginate hydrogels, allowing temporary retention of BMP-2 and other growth factors and reducing rapid outward diffusion. Growth-factor release was prolonged to approximately 3 weeks without an evident initial burst, while hydrogel degradation, swelling, and elastic modulus were only minimally affected (Jeon et al., 2011). This approach illustrates how carrier–cargo interactions can extend retention without requiring major changes to the bulk properties of the scaffold.

Taken together, these studies show that similar descriptions such as “sustained release” can arise from mechanistically distinct processes, including pore-dependent diffusion, carrier degradation, cargo localization, and affinity-based retention. Because carrier composition, loading conditions, and release assays differ among studies, release duration alone should not be used to rank delivery systems. The relevant criterion is whether a given mechanism generates an exposure profile appropriate for the therapeutic agent and biological context.

### 5.2 Simultaneous and sequential delivery

When multiple therapeutic agents are incorporated into the same regenerative strategy, their local exposure profiles may substantially overlap or be intentionally differentiated over time. Simultaneous delivery provides concurrent exposure to multiple signals, although the individual agents do not necessarily exhibit identical release kinetics. Sequential delivery, by contrast, is designed so that the major exposure periods of different agents occur at different times, often with some degree of overlap. The appropriate strategy should therefore be determined by the intended biological roles of the therapeutic agents rather than by an assumption that greater temporal complexity is inherently advantageous.

Simultaneous sustained delivery can be effective when the biological actions of two agents are intended to overlap. Li et al. incorporated rhBMP-2 and FGF-2 into chitosan nanocapsules combined with antigen-extracted xenogeneic cancellous bone and compared different release patterns in a rat intramuscular ectopic osteogenesis model. Among the regimens examined, simultaneous sustained release of rhBMP-2 and FGF-2 produced the greatest osteogenic response (Li et al., 2018). Although this ectopic model does not directly reproduce a bone-defect environment, the findings indicate that temporal separation is not necessarily required for effective dual-factor delivery.

When different therapeutic signals are expected to predominate at different stages, their release profiles can instead be temporally differentiated through carrier design. Min et al. encapsulated PDGF-BB and BMP-2 in separate microsphere formulations and incorporated both into a chitosan/glycerophosphate hydrogel. PDGF-BB was released earlier, whereas BMP-2 showed a delayed and more sustained release profile (Min et al., 2019). This system demonstrates that separate delivery compartments can be used to control the relative timing of factor release. However, the supporting evidence was based mainly on *in vitro* release and bioactivity assays and did not establish that this temporal pattern was superior to simultaneous delivery in a bone-defect model.

*In vivo* studies further demonstrate the feasibility of generating temporally differentiated exposure profiles within a single construct. Zhou et al. physically encapsulated FGF-2 within a gelatin methacryloyl (GelMA) hydrogel while binding BMP-2 to mineral-coated microparticles embedded in the same system. This configuration produced relatively rapid early FGF-2 release and more sustained BMP-2 release. Release was characterized over 28 days, with the early differences between FGF-2 and BMP-2 additionally quantified at days 1 and 7. In a rat critical-sized calvarial defect model, the dual-factor construct enhanced bone formation and was associated with increased angiogenic and osteogenic activity (Zhou et al., 2021). The hybrid dual-factor delivery concept and the differentiated release profiles of FGF-2 and BMP-2 are illustrated in Figure 6. Importantly, these findings support temporally differentiated release but do not establish that sequential delivery is universally superior to concurrent exposure.

**FIGURE 6:**
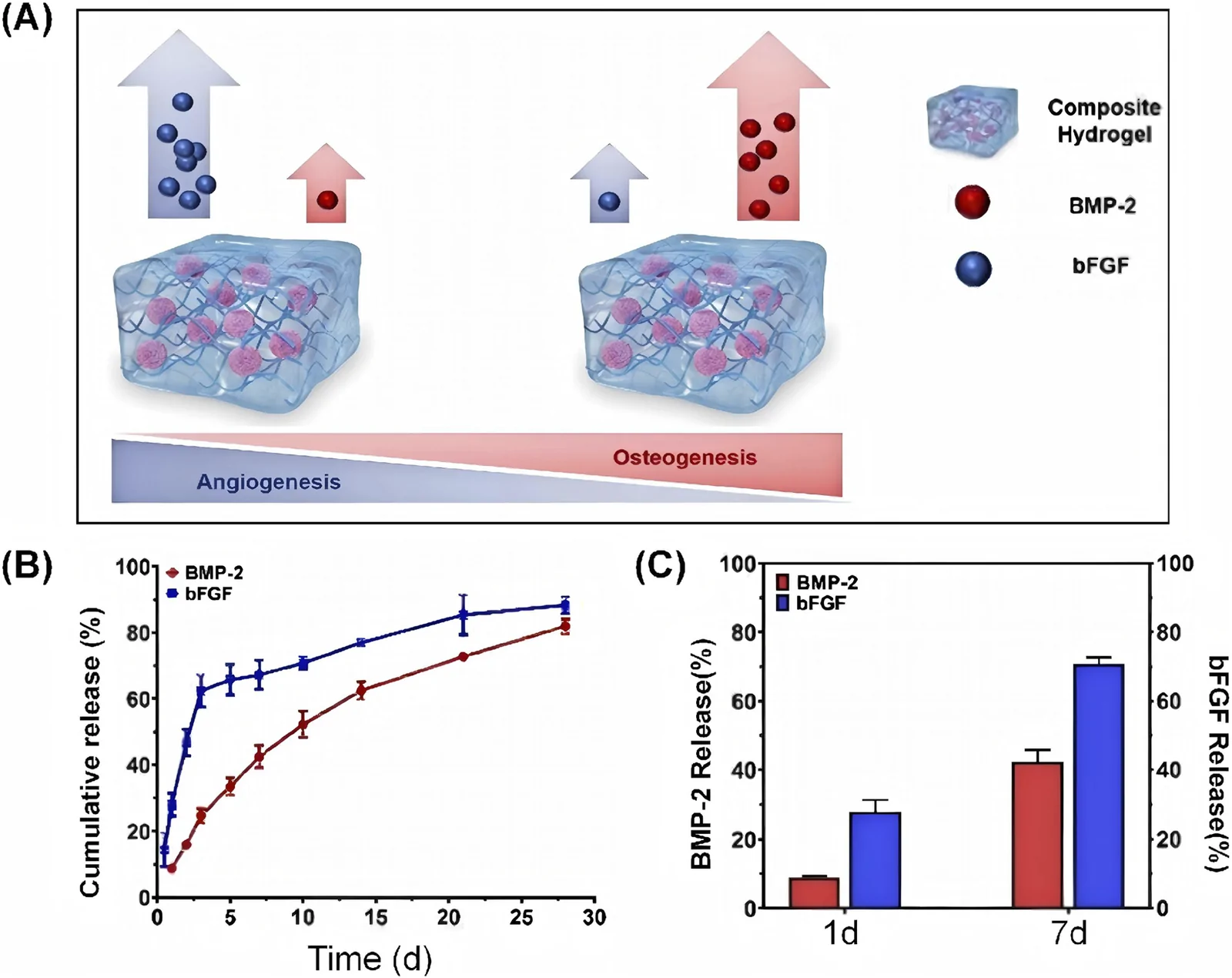
Differential temporal release of angiogenic and osteogenic growth factors from a hybrid dual-factor delivery system. **(A)** Schematic illustration of relatively early FGF-2 delivery and more sustained BMP-2 delivery. **(B)** Cumulative release profiles of BMP-2 and FGF-2 over 28 days. **(C)** Release percentages of BMP-2 and FGF-2 at 1 and 7 days. Adapted and rearranged from Scheme 1 and Figures 3D,E of the original study (Zhou et al., 2021). Source panels were cropped, resized, rearranged, and/or relabeled for the composite layout under the Creative Commons Attribution 4.0 International License (CC BY 4.0); no underlying experimental data were altered.

Simultaneous and sequential delivery should therefore be regarded as alternative design strategies rather than as different levels of sophistication. Concurrent exposure may be appropriate when therapeutic actions are intended to overlap, whereas temporally differentiated delivery may be useful when biological requirements change during repair. In either case, the relevant question is whether the resulting local exposure pattern improves regeneration relative to a simpler delivery strategy.

### 5.3 Stimuli-responsive release

The release strategies described above are largely predetermined by material structure, degradation, carrier–cargo interactions, and the spatial organization of therapeutic agents within the delivery system. The biological environment of a bone defect, however, changes during healing and may be further altered by infection, inflammation, oxidative stress, or other pathological conditions. Stimuli-responsive systems are designed to modify therapeutic release in response to such local changes or to externally applied physical stimuli. These approaches can therefore be broadly classified as endogenous- and exogenous-responsive systems (Cicuéndez et al., 2018; Zhang M. et al., 2024; Wu et al., 2025).

Endogenous-responsive systems use pathological features of the defect microenvironment as release triggers. A pH-sensitive scaffold developed by Cicuéndez et al. released levofloxacin relatively slowly at physiological pH but more rapidly under acidic conditions associated with bone infection (Cicuéndez et al., 2018). Enzyme activity can provide another trigger. Zhang et al. developed a matrix metalloproteinase (MMP)-responsive hydrogel in which increased MMP activity promoted phosphatidylserine release, modulated macrophage responses, and enhanced bone regeneration (Zhang et al., 2024a). The MMP-responsive PEG-pp-PS construct and its stimulus-dependent PS release behavior are illustrated in Figure 7A. Oxidative stress can likewise be exploited for responsive delivery. An injectable bone-adhesive hydrogel developed by Xing et al. released rutin in response to elevated reactive oxygen species and accelerated healing in a diabetic fracture model while suppressing excessive NLRP3 inflammasome activation (Xing et al., 2025). These systems demonstrate that local pathological signals can be coupled to therapeutic release, although the magnitude and timing of the response may vary among defects and during different stages of healing.

**FIGURE 7:**
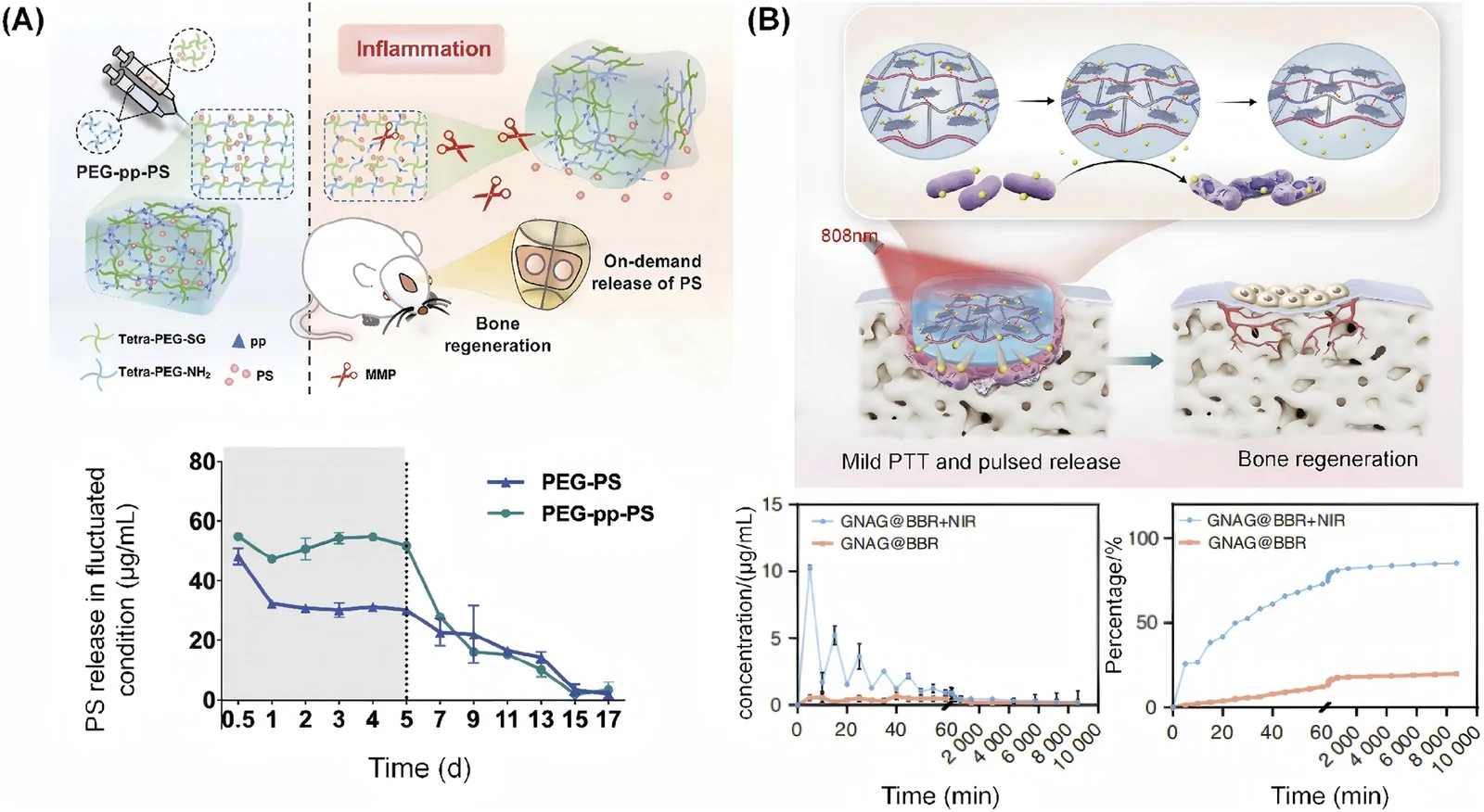
Representative endogenous- and exogenous-stimuli-responsive release strategies for local therapeutic delivery in bone regeneration. **(A)** Endogenous matrix metalloproteinase (MMP)-responsive delivery. The schematic shows the MMP-responsive PEG-pp-PS hydrogel, and the graph shows PS release under fluctuating MMP-2 conditions. Adapted and rearranged from Figures 1, 3F of the original study (Zhang M. et al., 2024). **(B)** Exogenous near-infrared (NIR)-triggered delivery. The schematic shows NIR-triggered pulsed berberine (BBR) delivery, and the graphs show intermittent and cumulative BBR release. Adapted and rearranged from Figures 1B, 4J,K of the original study (Wu et al., 2025). The two panels are derived from independent studies using different stimuli-responsive systems, experimental contexts, and release-assessment methods; they are presented as representative examples and are not intended for direct quantitative comparison. Source panels were cropped, resized, rearranged, and/or relabeled for the composite layout under the respective Creative Commons Attribution 4.0 International (CC BY 4.0) licenses; no underlying experimental data were altered.

Exogenous stimuli provide greater control over when a release-triggering signal is applied. Wang et al. incorporated black phosphorus nanosheets and strontium chloride into PLGA microspheres. Near-infrared (NIR) irradiation generated a photothermal effect that disrupted the PLGA shell and increased strontium-ion release, thereby enhancing bone regeneration in a rat femoral defect model (Wang et al., 2018). More recently, a photothermal-sensitive nanocomposite hydrogel was designed so that mild photothermal treatment triggered pulsed berberine release during infected bone-defect repair (Wu et al., 2025). The NIR-triggered mild-PTT strategy and the corresponding pulsed BBR release profiles are illustrated in Figure 7B. Externally triggered systems can therefore provide temporal control over stimulation, but their performance depends on factors such as tissue penetration, energy attenuation, and stimulus intensity.

Stimulus responsiveness should not, however, be equated with precise on-demand dosing. Endogenous signals such as pH, enzyme activity, and reactive oxygen species may vary both spatially and temporally within a defect, while the response obtained with an external stimulus may depend on tissue depth and local energy delivery. Release profiles established under controlled *in vitro* conditions may therefore differ substantially from those achieved *in vivo*. Evaluation should extend beyond demonstrating that a material responds to a stimulus and should determine how trigger magnitude relates to released dose, local therapeutic exposure, reproducibility, safety, and regenerative benefit.

Representative programmed and stimuli-responsive release strategies are summarized in Figures 6, 7, whereas a broader comparison of release-control approaches is provided in Table 3. Across these strategies, effective local delivery does not necessarily require slower release, longer duration, more complicated temporal programming, or greater material complexity. Release rate, duration, sequence, and responsiveness should instead be matched to the biological properties of the therapeutic agent and the changing requirements of bone repair.

**TABLE 3:** Representative strategies for controlling local therapeutic release in bone regeneration.

Release-control strategy	Representative implementation	Effect on release	Biological or regenerative relevance	Key delivery implication	References
Material architecture	Calcium sulfate dihydrate in BMP-2-loaded hydroxyapatite microspheres increases pore size and porosity after dissolution	Accelerates BMP-2 release by increasing diffusion	Enhanced BMP-2 delivery supports bone regeneration	Pore architecture directly regulates release rate; slower release is not inherently preferable	(Baek et al., 2018)
Carrier degradation	BMP-2 encapsulated in biodegradable PBVHx microspheres, with release coupled to polymer degradation	Sustained release during carrier degradation	Prolonged BMP-2 availability supports bone regeneration	Degradation kinetics should match the required exposure duration	(Zhang et al., 2025b)
Cargo localization within the carrier	BMP-2 preloaded into mesoporous hydroxyapatite nanoparticles rather than directly adsorbed onto the scaffold	Reduces initial burst and prolongs release	Improved local retention supports BMP-2 delivery and bone regeneration	Cargo location within the construct is an important release-control variable	(Qiu et al., 2020)
Affinity-based retention	Heparin immobilized in photocrosslinked alginate hydrogels to retain BMP-2 and other growth factors	Reduces rapid diffusion and prolongs release with little initial burst	Extends local growth-factor availability with minimal changes to bulk hydrogel properties	Reversible carrier–cargo interactions can reduce early loss and prolong exposure	(Jeon et al., 2011)
Multi-agent delivery	Simultaneous: rhBMP-2 and FGF-2 co-loaded into chitosan nanocapsules. Temporally differentiated: FGF-2 encapsulated in GelMA and BMP-2 bound to mineral-coated microparticles	Produces either overlapping sustained exposure or earlier release of one factor with more prolonged release of another	Both exposure patterns can support osteogenic or coordinated angiogenic–osteogenic responses	Release timing should match the biological roles of the agents; temporal separation is not inherently superior	(Li et al., 2018; Zhou et al., 2021)
Stimuli-responsive release	Endogenous: acidic pH, MMP activity, or ROS regulate levofloxacin, phosphatidylserine, or rutin release. Exogenous: NIR stimulation increases Sr^2+^ release or triggers pulsed berberine release	Adjusts release in response to pathological cues or externally applied stimulation	Supports antimicrobial activity, immunomodulation, or bone repair depending on the system	Responsiveness can adapt release to local or external cues but does not guarantee precise dosing	(Cicuéndez et al., 2018; Zhang et al., 2024a; Wu et al., 2025; Xing et al., 2025)

Abbreviations: BMP-2, bone morphogenetic protein-2; GelMA, gelatin methacryloyl; MMP, matrix metalloproteinase; NIR, near-infrared; PBVHx, poly (3-hydroxybutyrate-co-3-hydroxyvalerate-co-3-hydroxyhexanoate); rhBMP-2, recombinant human bone morphogenetic protein-2; ROS, reactive oxygen species.

## 6 Tailored delivery strategies for challenging bone defects

Bone defects with similar dimensions may have substantially different reasons for impaired healing. Critical-sized defects are characterized by markedly limited intrinsic regenerative capacity and may involve extensive tissue loss, inadequate vascularization, and insufficient recruitment of reparative cells (Fang et al., 2024). Infected defects additionally require control of residual microorganisms, biofilms, and persistent inflammation (Zhang H. et al., 2025), whereas osteoporotic and diabetes-associated defects develop within abnormal remodeling or metabolic environments (Cai et al., 2023; Zhang X. et al., 2024). Therapeutic agents, carrier properties, and release profiles should therefore be selected according to the dominant biological barriers of each defect rather than applied uniformly across different pathological settings. Antimicrobial, metabolic, and antiresorptive agents are discussed only as disease-specific adjuncts when their local delivery addresses pathological barriers that constrain the immunoregulatory, angiogenic, or osteogenic processes central to this review.

### 6.1 Critical-sized bone defects

Critical-sized bone defects (CSDs) are generally defined by their inability to heal spontaneously under specified experimental conditions and observation periods. The threshold for a critical defect is not universal, however, and varies with species, anatomical site, defect geometry, and experimental design. Studies using rabbit calvarial and mandibular models have further shown that critical dimensions need to be established for specific anatomical sites (Kotagudda Ranganath et al., 2022). Defect size alone also does not fully predict clinical healing. In the SPRINT trial, some tibial defects meeting predefined critical-size criteria healed without additional surgical intervention, indicating that size is an important but not exclusive determinant of outcome (Sanders et al., 2014). CSDs are therefore better understood as defects with markedly limited intrinsic healing capacity than as lesions defined by a single universal dimensional threshold.

Limited healing in CSDs reflects not only the extent of tissue loss but also disruption of the local regenerative environment. Single-cell RNA sequencing by Fang et al. identified fewer progenitor and endothelial cells together with increased pro-inflammatory and fibrotic cell populations in CSDs compared with smaller defects. Reduced expression of *Spp1* and *Cxcl12* was also observed, and biomaterial-mediated delivery targeting these deficient signals promoted vascular restoration and bone regeneration (Fang et al., 2024). A complementary study showed that enhancing endothelial progenitor-cell angiogenic activity improved vascularization and bone repair in a critical-sized defect model (Cao et al., 2024). These findings indicate that therapeutic selection in CSDs should address the specific cellular and vascular deficits within the regenerative environment rather than relying solely on stronger osteogenic stimulation.

Maintaining adequate local therapeutic exposure across a large defect is another important consideration. Kolambkar et al. delivered BMP-2 from an alginate hydrogel contained within a perforated nanofiber mesh tube in a rat segmental femoral defect. Compared with collagen-sponge delivery at the same BMP-2 dose, this system improved local BMP-2 delivery and resulted in greater bone formation and functional recovery (Kolambkar et al., 2011). More recently, a bilayer scaffold prolonged BMP-2 release relative to a collagen sponge and increased local exposure within the defect, thereby improving repair of critical-sized calvarial defects (Kim et al., 2024). For CSDs, effective delivery therefore requires both identification of the principal biological deficits and sufficient local exposure to the selected therapeutic signals during the relevant stages of regeneration.

### 6.2 Infected bone defects

Infected bone defects require simultaneous consideration of pathogen control and tissue regeneration. Even after surgical debridement, residual bacteria and biofilms may sustain infection and inflammation and further compromise the regenerative microenvironment (Zhang H. et al., 2025). Local antimicrobial delivery can increase drug exposure at the affected site, but the objective should be sufficient infection control without unnecessarily prolonging antimicrobial exposure into later stages of repair.

The balance between antimicrobial activity and bone regeneration depends on both the carrier and the locally delivered drug. Vancomycin incorporated into gelatin/β-tricalcium phosphate scaffolds showed release and regenerative outcomes that varied with scaffold composition in a rabbit chronic osteomyelitis model, with an intermediate β-tricalcium phosphate content providing the best overall balance between infection control and new bone formation (Zhou et al., 2018). Drug-related effects on regeneration also require consideration. In noninfected rat mandibular defects, local vancomycin reduced bone formation, whereas combined vancomycin and tobramycin did not produce the same degree of inhibition (Han et al., 2021). Although this noninfected model does not directly reproduce an infected defect, the findings indicate that local antimicrobial therapy should be evaluated not only for pathogen control but also for drug selection, dose, and potential effects on bone formation.

Regenerative support can be incorporated alongside antimicrobial treatment. Co-delivery of lysostaphin and BMP-2 from a hydrogel reduced *Staphylococcus aureus* infection while supporting bone regeneration in infected segmental defects (Johnson et al., 2019), demonstrating that antibacterial and osteogenic functions can be integrated within a single delivery platform. Alternatively, therapeutic exposure can be differentiated over time when infection control and bone regeneration have different temporal requirements. Zheng et al. incorporated vancomycin into an injectable thermosensitive hydrogel and embedded microspheres loaded with the collagen-derived Asp-Gly-Glu-Ala (DGEA) peptide within the same construct. Vancomycin was preferentially released during hydrogel degradation, whereas the more slowly degrading microspheres subsequently provided sustained DGEA release to support bone formation (Zheng et al., 2024). Infected bone defects may therefore benefit from effective early antimicrobial exposure combined with concurrent or subsequent regenerative support, rather than prolonged high-intensity antimicrobial treatment throughout the entire repair period.

### 6.3 Osteoporotic bone defects

Osteoporotic bone defects heal within an abnormal remodeling environment characterized by reduced bone formation and excessive or dysregulated bone resorption, often accompanied by oxidative stress and persistent inflammation (Luo et al., 2025). Effective local treatment may therefore require more than direct osteogenic stimulation. Zhang et al. incorporated human fetal mesenchymal stem cell secretome into a gelatin/acrylated β-cyclodextrin host–guest hydrogel, which promoted cell spreading and osteogenic differentiation, recruited endogenous integrin β1-positive cells, and increased new bone formation and bone strength in osteoporotic rats (Zhang X. et al., 2024). This study illustrates how local delivery can support regeneration by improving the microenvironment in which reparative cells function.

Release behavior is also important in osteoporotic defects because therapeutic signals may need to be maintained over extended periods. Xu et al. encapsulated parathyroid hormone (1–34) in PLGA microspheres, incorporated the microspheres together with simvastatin into a GelMA hydrogel, and reinforced the construct with a 3D-printed polylactic acid scaffold. PLGA encapsulation reduced the initial release of the peptide, which was subsequently released for approximately 40 days, while dual delivery enhanced bone formation in osteoporotic rat calvarial defects (Xu et al., 2024). Hayashi et al. incorporated an anti-sclerostin antibody into carbonate-apatite honeycomb granules, with release varying according to local pH and resulting in improved bone quantity and remodeling in ovariectomized rabbit femoral defects (Hayashi et al., 2024). These studies demonstrate that therapeutic selection and release kinetics need to be considered together in osteoporotic bone regeneration.

Carrier properties and local drug exposure can substantially alter regenerative outcomes. VEGF-loaded crystalline silicon-substituted hydroxyapatite scaffolds promoted vascularization and bone formation in osteoporotic sheep, whereas nanocrystalline scaffolds were associated with greater fibrous tissue formation and inflammation (Casarrubios et al., 2020). A zoledronic acid-loaded ceramic coating on 3D-printed biodegradable magnesium alloy scaffolds slowed scaffold degradation and drug release while improving new bone formation in osteoporotic defects (Ran et al., 2023). By contrast, within another osteoporotic sheep model, incorporation of 1 wt% zoledronic acid into mesoporous bioactive glass/PCL scaffolds was associated with osteoblast-like cell apoptosis, suppressed osteoclast differentiation, increased inflammation, and impaired healing (Gómez-Cerezo et al., 2019). These contrasting findings reinforce the importance of controlling carrier properties, local dose, and release duration rather than assuming that greater local drug exposure will necessarily improve osteoporotic bone repair. Local biomaterial-based therapy should therefore be regarded as an adjunct for improving the regenerative environment at the defect site rather than as a substitute for systemic management of osteoporosis.

### 6.4 Diabetic bone defects

Diabetic bone regeneration is impaired by multiple interacting abnormalities, including reduced osteogenic capacity, hyperglycemia-associated stress, oxidative damage, and impaired angiogenesis. In a type 2 diabetic rat distraction-osteogenesis model, Cai et al. observed slower bone formation, reduced bone quantity, poorer mechanical properties, and lower HIF-1α and VEGF expression than in nondiabetic controls (Cai et al., 2023). Although this model does not represent a conventional bone defect, it illustrates how diabetes can compromise both tissue formation and vascular support during skeletal regeneration. Therapeutic strategies should therefore address the broader diabetic microenvironment rather than focus exclusively on osteogenic stimulation.

Selection of the therapeutic agent should reflect the specific regenerative processes impaired by diabetes. Wallner et al. compared local VEGF-A and fibroblast growth factor-9 (FGF-9) treatment in type 2 diabetic mice and found that FGF-9 produced greater restoration of angiogenesis, osteogenesis, and bone remodeling, whereas VEGF-A achieved only partial recovery (Wallner et al., 2015). This finding indicates that simply providing a pro-angiogenic factor does not guarantee equivalent regenerative benefit. Metabolic modulators can also serve as disease-specific adjuncts by improving the local diabetic microenvironment that constrains regeneration. Local delivery of metformin from a 3D-printed GelMA–nanoclay hydrogel scaffold enhanced immunomodulatory, angiogenic, osteogenic, and regenerative responses in diabetic bone defects (Li H. et al., 2024). Thus, therapeutic selection in diabetic defects need not be limited to conventional osteogenic growth factors.

Carrier composition and release behavior can also be adapted to the diabetic microenvironment. A 3D-printed alginate/nanohydroxyapatite/small-intestinal-submucosa scaffold provided structural support while improving immunoregulatory, angiogenic, and osteogenic responses in diabetic bone defects (Tan et al., 2024). More active microenvironment-responsive designs have also been developed. Fu et al. constructed a glucose/ROS-responsive hydrogel containing a cerium–zoledronic acid–glucose oxidase nanozyme. The system reduced local glucose and reactive oxygen species, attenuated inflammation, promoted angiogenesis and osteogenesis, and subsequently released zoledronic acid to suppress excessive osteoclast activity, thereby improving diabetic calvarial-defect repair (Fu et al., 2025). This approach illustrates how pathological features of diabetes can be incorporated directly into the logic of local delivery-system design.

Local treatment, however, cannot eliminate the underlying systemic metabolic disorder. Biomaterial-based delivery should therefore be viewed as a means of correcting local regenerative deficits rather than as a substitute for systemic glycemic control. Evaluation of diabetic bone repair should consequently consider not only new bone formation but also vascularization, bone quality, mechanical recovery, and the metabolic context in which regeneration occurs. The dominant barriers to healing and corresponding therapeutic and delivery priorities across the challenging bone-defect conditions discussed in this section are summarized in Table 4.

**TABLE 4:** Pathological barriers and tailored local-delivery priorities in challenging bone defects.

Defect condition	Dominant barriers to healing	Therapeutic priority	Local-delivery priority	Key consideration	References
Critical-sized bone defects	Limited intrinsic healing, extensive tissue loss, insufficient vascularization and cell recruitment, and inflammatory or fibrotic alterations	Restore vascular and cellular regenerative capacity while providing osteogenic support	Maintain adequate exposure across the defect while targeting impaired vascular, cellular, and osteogenic processes	Criticality varies by site and model; osteogenic stimulation alone may not correct multiple regenerative deficits	(Sanders et al., 2014; Kotagudda Ranganath et al., 2022; Fang et al., 2024)
Infected bone defects	Residual microorganisms and biofilms, persistent infection and inflammation, and impaired bone formation	Control infection while creating conditions favorable for regeneration	Provide sufficient early antimicrobial exposure with concurrent or subsequent regenerative support	Evaluate both infection control and regeneration; drug choice, local dose, and exposure duration are important	(Zhou et al., 2018; Zheng et al., 2024; Zhang et al., 2025a)
Osteoporotic bone defects	Reduced bone formation, dysregulated bone resorption, and abnormal remodeling, often with oxidative stress and inflammation	Restore a favorable balance between bone formation and resorption	Match the agent, carrier, dose, and release profile to the remodeling deficit	Greater local exposure is not necessarily beneficial; excessive antiresorptive exposure may impair repair	(Gómez-Cerezo et al., 2019; Xu et al., 2024; Luo et al., 2025)
Diabetes-associated bone defects	Hyperglycemia-related stress, oxidative damage, persistent inflammation, impaired angiogenesis, and reduced osteogenic capacity	Improve the metabolic and inflammatory microenvironment while restoring vascular and osteogenic function	Target glucose- and reactive oxygen species-related damage together with regenerative deficits; pathological cues may guide responsive release	Local delivery cannot correct systemic hyperglycemia and should complement metabolic control	(Cai et al., 2023; Tan et al., 2024; Fu et al., 2025)

## 7 Evaluation of delivery performance and regenerative efficacy

Whereas Section 5 focuses on how biomaterial and delivery-system design can regulate release profiles, this section evaluates whether the intended release behavior is actually achieved and whether it translates into biologically meaningful local therapeutic exposure. Evaluation of a local delivery system should extend beyond its nominal loading dose, *in vitro* release profile, or the amount of newly formed bone. A meaningful assessment should determine how the therapeutic cargo is released, whether biological activity is retained after formulation and release (Olthof et al., 2018; Koons et al., 2021; Gómez-Lázaro et al., 2024), whether the intended biological deficit is corrected, and whether this response ultimately results in structurally and functionally relevant regeneration (Priddy et al., 2023). The experimental model and reporting framework are equally important because apparent efficacy can depend strongly on model design and methodological rigor (Percie du Sert et al., 2020). Delivery performance should therefore be evaluated across multiple levels, from release and bioactivity to biological response, tissue repair, mechanical recovery, and reproducibility.

### 7.1 Release characterization and retained bioactivity

The amount of therapeutic cargo incorporated into a delivery system does not necessarily correspond to the amount subsequently released. Release characterization should therefore distinguish loading from release and report parameters such as the initial burst, cumulative release, and duration of release. Interpretation of these measurements also requires attention to experimental conditions. *In vitro* release profiles can vary with the testing method and release environment, while standardized protocols for nano- and microcarrier systems remain limited (Gómez-Lázaro et al., 2024). Release percentage or duration should therefore not be interpreted independently of the method used to generate these measurements.

*In vitro* release profiles also cannot be assumed to reproduce therapeutic exposure after implantation. Olthof et al. showed that modifying the *in vitro* release environment substantially altered measured BMP-2 release and that *in vitro* models had limited ability to predict the *in vivo* behavior of different delivery formulations (Olthof et al., 2018). *In vitro* testing remains essential for comparing formulations and characterizing release mechanisms, but its relationship to *in vivo* release and local exposure should be established whenever possible.

Quantifying released protein is also insufficient if biological activity has been compromised during material processing, storage, or release. Koons et al. found that high-temperature processing did not completely prevent BMP-2 release but reduced the amount of bioactive BMP-2 recovered from the resulting constructs (Koons et al., 2021). The method used to assess bioactivity also matters. Fung et al. showed that cellular assays differed in their sensitivity to rhBMP-2-induced alkaline phosphatase activity (Fung et al., 2019). Delivery studies should therefore evaluate both the quantity of therapeutic cargo released and the biological activity retained after release, rather than assuming that measured concentration is equivalent to effective therapeutic exposure.

### 7.2 Biological response and tissue regeneration

Regenerative efficacy should not be inferred solely from the amount of newly formed bone. Evaluation should first determine whether the delivery system modifies the biological process it was designed to target. Immunomodulatory systems should assess relevant inflammatory and immune responses, whereas angiogenic strategies should evaluate vascular formation, perfusion, or other measures of vascular function. Han et al., for example, showed that early changes in local blood flow predicted subsequent femoral graft healing in mice, demonstrating that early biological responses can provide information that is not captured by final bone measurements alone (Han et al., 2018). Mechanistic or surrogate endpoints, such as macrophage-marker expression, cell migration, endothelial tube formation, or osteogenic-marker expression, should therefore be interpreted as evidence of biological activity rather than as direct evidence of complete structural or functional bone regeneration.

Structural regeneration within the defect should then be quantified. Micro-computed tomography (micro-CT) provides valuable three-dimensional assessment of mineralized tissue, but quantitative outcomes can be affected by scan parameters, segmentation thresholds, and the selected region of interest. These methodological details should therefore be reported clearly (Bouxsein et al., 2010). Bone volume should also be interpreted together with defect bridging and the spatial distribution of regenerated tissue. A construct that produces substantial mineralized tissue at the defect margins but leaves the central defect unrepaired should not be considered equivalent to complete defect regeneration.

Imaging alone cannot fully characterize the biological quality of the regenerated tissue. Histological analysis can identify fibrous tissue, residual biomaterial, persistent inflammatory responses, and the interface between newly formed bone and the implanted material. Standardized histomorphometric terminology and measurement approaches can further improve consistency and comparability across studies (Dempster et al., 2013). Regenerative efficacy should therefore be evaluated by integrating evidence for the intended biological response with the quantity, distribution, bridging, and histological quality of newly formed tissue.

### 7.3 Functional and mechanical recovery

Greater bone formation does not necessarily indicate restoration of mechanical competence. Priddy et al. showed that changes in bone quantity and spatial distribution were not accompanied by proportional changes in mechanical performance, demonstrating that imaging outcomes alone cannot establish whether regenerated bone has recovered sufficient strength (Priddy et al., 2023).

Mechanical testing is therefore particularly important for load-bearing long-bone defects. Depending on anatomical location and the dominant loading mode, bending, compression, or torsional testing can be used to characterize stiffness, strength, and load-bearing capacity. De La Vega et al. evaluated segmental femoral defects using both micro-CT and torsional testing and demonstrated the importance of mechanical assessment in determining whether regenerated bone approached the functional properties of intact bone (De La Vega et al., 2022). Successful regeneration should therefore be defined not only by mineralized tissue formation but also by stable defect bridging and recovery of mechanical function.

### 7.4 Model relevance, reproducibility, and reporting

The apparent efficacy of a delivery system depends in part on whether the experimental model reproduces the biological condition that the therapy is intended to treat. Antimicrobial strategies, for example, should be evaluated in models that reproduce relevant features of bone infection rather than inferred solely from sterile defects. Osteomyelitis models differ in methods of disease induction, animal species, and the clinical settings they are intended to represent, and model selection should therefore be guided by the specific research question (Huang et al., 2023). Similar considerations apply to critical-sized defects, for which the threshold for spontaneous healing varies with species, age, anatomical site, and defect characteristics rather than being defined by a single universal dimension (Wei et al., 2024).

Different models address different levels of evidence and should not be interpreted interchangeably. *In vitro* studies characterize release, bioactivity, and mechanisms but do not establish defect repair; ectopic models demonstrate osteogenic potential outside the native bone environment; calvarial defects provide orthotopic but largely non-load-bearing evidence; and segmental long-bone models better address fixation and mechanical recovery. Preclinical long-bone studies nevertheless remain highly heterogeneous in defect definition, fixation, and follow-up (Maai et al., 2026). Human evidence, discussed in Section 8.1, remains limited for advanced local-delivery systems.

Methodological rigor is equally important. Appropriate controls, sample-size justification, randomization, blinding where feasible, predefined exclusion criteria, and transparent reporting reduce the risk that apparent treatment effects arise from experimental bias. The ARRIVE 2.0 guidelines provide a framework for improving the design and reporting of animal research (Percie du Sert et al., 2020). Convincing evidence for a local delivery system therefore requires not only favorable regenerative outcomes but also a biologically relevant model, rigorous study design, and sufficient methodological transparency to permit interpretation and reproduction of the findings.

## 8 Discussion and future perspectives

Biomaterial-mediated local delivery provides opportunities to increase therapeutic retention, regulate local exposure, and coordinate treatment with the biological requirements of bone repair. The evidence reviewed above, however, also shows that improved preclinical regeneration does not necessarily imply greater clinical value. Multi-agent, long-acting, sequential, and stimuli-responsive systems may offer advantages when they address specific biological limitations, but they can also increase manufacturing complexity, quality-control requirements, safety concerns, and regulatory burden (Spiller and Vunjak-Novakovic, 2015; Herczeg and Song, 2022). The central translational question is therefore not whether a delivery system can be made more sophisticated, but whether its added features produce a reproducible therapeutic benefit that justifies the additional complexity. Clinical development should consequently consider therapeutic exposure, safety, manufacturability, and practical implementation together rather than optimizing regenerative efficacy in isolation.

### 8.1 Barriers to clinical translation

Safety is a fundamental requirement for clinical translation. Local administration can increase therapeutic exposure at the defect site, but it is not inherently safer than systemic administration or conventional delivery. Excessive local rhBMP-2 exposure, rapid initial release, and diffusion into surrounding tissues have been associated with inflammatory responses and ectopic bone formation (Chernysheva et al., 2025). Clinical development should therefore define a therapeutic window that provides sufficient regenerative activity while limiting adverse effects, rather than relying on progressively higher loading doses or longer release periods.

Reproducible manufacturing is another major barrier. A delivery system that performs well in laboratory-scale preparation must retain comparable architecture, mechanical properties, degradation behavior, cargo loading, and release characteristics after scale-up. Sterilization adds further difficulty because commonly used sterilization methods can alter biodegradable materials, while systems carrying proteins or other labile therapeutics must also preserve cargo bioactivity (Herczeg and Song, 2022; Khodabukus et al., 2022). Manufacturing, sterilization, storage, and shelf stability should therefore be considered during early system development rather than addressed only after preclinical efficacy has been demonstrated.

Increasing system complexity can further complicate translation. Each additional therapeutic agent, material component, or responsive mechanism may introduce new manufacturing steps, analytical requirements, and regulatory considerations. This challenge is particularly relevant to controlled protein-delivery systems, in which both the therapeutic component and the material carrier must be characterized and controlled (Spiller and Vunjak-Novakovic, 2015). Complex designs should therefore be justified by therapeutic advantages that cannot be achieved with a simpler formulation.

Human validation remains the final and most important requirement. Most advanced biomaterial-based local-delivery systems discussed in this review remain at the preclinical stage. By contrast, clinical evaluation of FGF-2 delivery using gelatin hydrogel has been reported in lower-limb fracture, knee osteotomy, and osteonecrosis of the femoral head (Kuroda et al., 2019). Broader clinical adoption, however, will require not only evidence of efficacy and safety but also practical considerations such as storage, intraoperative handling, and compatibility with established surgical procedures.

### 8.2 Future perspectives

Future research should focus on three testable priorities: defining dose–exposure relationships, demonstrating added benefit over simpler delivery systems, and improving the predictive relevance of preclinical models. The administered or loaded dose alone does not determine regenerative efficacy because different carriers can produce substantially different retention and release behavior. Boerckel et al. showed in an 8-mm rat femoral segmental defect that the rhBMP-2 dose required for effective bone regeneration depended on the delivery system: at the same 1.0 μg dose, a nanofiber mesh/alginate system achieved 100% defect bridging and approximately 2.5-fold greater bone volume than collagen-sponge delivery by 12 weeks, consistent with greater local protein retention (Boerckel et al., 2011). Vantucci et al. further showed in an 8-mm chronic rat femoral nonunion model that 30 μg BMP-2 delivered using heparin microparticles within an alginate/PCL construct exhibited sustained *in vitro* release over 4 weeks with <10% initial release during the first 6 h, while increasing total bone volume and reducing peak heterotopic ossification compared with collagen-sponge delivery of the same dose (Vantucci et al., 2021). Future studies should therefore characterize the relationships among loading dose, actual release, local exposure, biological response, and final regeneration rather than comparing formulations on the basis of dose or release duration alone.

Greater material sophistication should likewise not be treated as an endpoint in itself. Programmable biomaterials can generate sequential or environment-responsive release profiles and may be valuable when different stages of repair require different interventions (Song et al., 2024). Multi-agent delivery, temporal programming, and stimulus responsiveness, however, are not inherently superior to simpler approaches. Their value should be established through direct comparison with appropriate simpler controls to determine whether additional materials, therapeutic agents, or responsive functions provide a meaningful biological or functional advantage.

Improved preclinical models may also help identify delivery strategies that warrant further development. Conventional two-dimensional cell cultures cannot reproduce the full multicellular and three-dimensional environment of bone, whereas animal studies are affected by species- and model-specific differences. Bone-related organoids offer more tissue-relevant platforms for mechanistic investigation, drug screening, and biomaterial evaluation, although limitations in vascularization, maturation, and standardization currently favor their use as complements rather than replacements for existing models (Bai et al., 2024). Computational approaches provide another complementary tool. Perier-Metz et al. used large-animal bone-defect data to develop an *in silico* model predicting how scaffold design and material properties influence bone formation (Perier-Metz et al., 2022). Such approaches may help prioritize candidate systems before extensive *in vivo* testing and reduce dependence on empirical trial-and-error.

### 8.3 Limitations of this review

As a narrative review, this article was not based on a prespecified systematic-review protocol, and literature selection may therefore be susceptible to some degree of selection bias. In addition, substantial heterogeneity across studies in animal species, defect sites and sizes, therapeutic doses, carrier designs, release-assessment methods, and follow-up periods limits direct cross-study comparison and precludes quantitative synthesis. These limitations should be considered when interpreting the relative performance and translational potential of different delivery strategies.

## 9 Conclusion

Biomaterial-mediated local delivery can improve therapeutic retention and regulate local exposure during bone repair, but successful regeneration depends on matching therapeutic agents, carriers, doses, and release profiles to the dominant biological barrier and stage of healing. Because inflammatory dysregulation, impaired vascularization, insufficient cell recruitment, and reduced osteogenic capacity vary among critical-sized, infected, osteoporotic, and diabetes-associated defects, no single delivery strategy is likely to be optimal across all settings.
